# A machine learning model exploring the relationship between chronic medication and COVID-19 clinical outcomes

**DOI:** 10.1007/s11096-025-01955-7

**Published:** 2025-07-28

**Authors:** Berta Miró, Natalia Díaz González, Juan-Francisco Martínez-Cerdá, Clara Viñas-Bardolet, Alex Sánchez-Pla, Adrián Sánchez-Montalvá, Marta Miarons

**Affiliations:** 1 Statistics and Bioinformatics Unit, Vall d’Hebron Institut de Recerca (VHIR), Barcelona, Spain; 2 Agency for Health Quality and Assessment of Catalonia (AQuaS), Barcelona, Spain; 3 Genetics, Microbiology and Statistics Department, Universitat de Barcelona, Barcelona, Spain; 4 Centro de Investigación Biomédica en Red Fragilidad y Envejecimiento Saludable, Instituto de Salud Carlos III, 28029 Madrid, Spain; 5 Department of Medicine, Universitat Autònoma de Barcelona, Barcelona, Spain; 6 International Health Unit Vall d’Hebron (PROSICS), Infectious Diseases Department, Vall d’Hebron University Hospital, Vall d’Hebron Institute of Research, Barcelona, Spain; 7 Centre for Biomedical Research in Infectious Diseases Network (CIBERINFEC), Institute of Health Carlos III, Madrid, Spain; 8 Pharmacy Department, Vall d’Hebron Hospital Universitari, Vall d’Hebron Barcelona Hospital Campus, Barcelona, Spain; 9 Pharmacy Department, Consorci Hospitalari de Vic, Barcelona, Spain

**Keywords:** ACE inhibitors, ARBs, COVID-19, HMG-CoA reductase, Machine learning, Metformin, Mortality, Polypharmacy, Prediction models

## Abstract

**Background:**

The impact of chronic medication on COVID-19 outcomes has been a topic of ongoing debate since the onset of the pandemic. Investigating how specific long-term treatments influence infection severity and prognosis is essential for optimising patient management and care.

**Aim:**

This study aimed to investigate the association between chronic medication and COVID-19 outcomes, using machine learning to identify key medication-related factors.

**Method:**

We analysed 137,835 COVID-19 patients in Catalonia (February–September 2020) using eXtreme Gradient Boosting to predict hospitalisation, ICU admission, and mortality. This was complemented by univariate logistic regression analyses and a sensitivity analysis focusing on diabetes, hypertension, and lipid disorders.

**Results:**

Participants had a mean age of 53 (SD 20) years, with 57% female. The best model predicted mortality risk in 18 to 65-year-olds (AUCROC 0.89, CI 0.85–0.92). Key features identified included the number of prescribed drugs, systemic corticoids, 3-hydroxy-3-methylglutaryl coenzyme A (HMG-CoA) reductase, and hypertension drugs. A sensitivity analysis identified that hypertensive participants over 65 taking angiotensin-converting enzyme (ACE) inhibitors or angiotensin II receptor blockers (ARBs) had lower mortality risk (OR 0.78 CI 0.68–0.92) compared to those on other antihypertensive medication (OR 0.8 CI 0.68–0.95). Treatment with inhibitors of dipeptidyl peptidase 4 was associated to higher mortality in participants aged 18–65, while metformin showed a protective effect in those over 65 (OR 0.79, 95% CI 0.68–0.92).

**Conclusion:**

Machine learning models effectively distinguished COVID-19 outcomes. Patients under ACEi or ARBs or biguanides should continue their prescribed medications, which may offer protection over alternative treatments.

**Supplementary Information:**

The online version contains supplementary material available at 10.1007/s11096-025-01955-7.

## Impact statements

- Machine learning-driven models effectively predict hospitalisation, ICU admission, and death in COVID-19 patients.
- ACEi, ARBs, and biguanides (metformin) demonstrated potential protective effects.
- Findings support continued use of these medications without switching to alternatives.
- The study methodology is scalable and can be applied at a low additional cost using AI-driven analysis of administrative health databases.

## Introduction

COVID-19 emerged as an unprecedented public health crisis, placing immense strain on national health systems. Globally, the outbreak disrupted economies, hindered trade, and restricted mobility [1], created challenges in medication supply, and pharmacy services [2].

Various studies have been conducted to determine risk factors associated with poor COVID-19 outcomes [3–8], although a reduced number used health record data [9–13]. While unmodifiable factors like age, sex, and comorbidities have been linked to severe COVID-19 outcomes [14, 15], potentially modifiable factors such as chronic medication use have also drawn considerable attention. Since the first wave, studies have examined how long-term treatments [16–21], particularly cardiovascular drugs like angiotensin-converting enzyme (ACE) inhibitors and angiotensin II receptor blockers (ARBs), may influence disease severity. Evidence suggests there is no increase in mortality risk [22–24]. Corticosteroids and other anti-inflammatories have also been used in managing COVID-related respiratory distress [25].

The management of chronic conditions and associated medications has been a key focus [26], emphasizing the importance of effective disease control with drugs that either avoid harmful interactions or offer protection against COVID-19, thereby reducing potential complications [27].

### Aim

This study aimed to investigate the association between chronic medication and COVID-19 outcomes, using machine learning to identify key medication-related factors.

### Ethics approval

This study obtained ethical clearance from the Ethics Committee from Vall d’Hebrón in 2020 (reference number: PR (AG) 240/2020) and received approval through a public call for grants to utilize the PADRIS databases in research projects.

## Method

### Study population, registry features, and data acquisition

The Data Analytics Program for Research and Innovation in Health (PADRIS) provided the data used in this study, drawing administrative data primarily from the Electronic Health System of Catalonia, centralized by the Agency for Health Quality and Assessment of Catalonia (AQuaS). This study was not in any clinical trial registry.

The final PADRIS pseudonymised database was built by integrating six administrative databases: patient demographics and socioeconomic data, COVID-19 diagnoses, comorbidities, drugs dispensed by community pharmacies, drugs dispensed by hospital pharmacies, and ICU admissions. It included 138,218 individuals diagnosed with COVID-19 in Catalonia between February 27 and September 14, 2020.

Medical diagnosis and comorbidities adhered to the International Statistical Classification of Diseases and Related Health Problems 10th Edition (ICD-10) [28]. The pharmacy invoicing data followed the Anatomical Therapeutic Chemical classification established by the World Health Organization Collaborating Centre (Supplementary Material 1) [29]. Chronic medication use was defined as a drug dispensed from the community or hospital pharmacy for at least 30 days prior to COVID-19 diagnosis, with continued use at diagnosis. For patients that initiated a medication within 30 days of diagnosis, that exposure was classified as non-use and excluded, consistent with previous literature [30, 31].

SARS-CoV-2 infection was identified via positive nucleic acid amplification tests, approved rapid antigen tests or serology tests indicating recent infection, or clinical symptoms with close epidemiological links to confirmed cases [32].

The Adjusted Morbidity Groups (GMA) categorised the population into five groups attributed to the risk of hospital admission and overall survival, and were used for comorbidity risk assessment [33]. The socioeconomic status information classified individuals into four groups based on annual gross income.

### Data processing

Data pre-processing was performed using Pentaho Data Integration 9.0.0.0–423. During extraction, missing or inconsistent data, non-study participants, and duplicates were identified and corrected, or removed. Participants missing GMA values (n = 7089) were excluded, with no significant impact on variable distribution. Transformation steps included homogenisation of formats, unifying ICD or ATC nomenclatures (Supplementary Material 1), and generating binary variables for drugs and diseases. Comorbidities were cross-validated against administered medications, revealing under-registration of HIV, which was corrected by reclassifying participants on HIV-specific treatments. No data imputation was performed. In the load phase, data were de-identified and integrated into a unified dataset. Privacy measures included numeric value replacement and removal of variables with fewer than 10 participants. The resulting database was reviewed by the data protection committee before use.

### Statistical analyses

Three main analyses were conducted. First, a univariate descriptive analysis was performed for all variables, including comparative analyses of potential predictive factors, stratified by age and sex. Next, prediction models for each COVID-19 outcome considered (hospitalisation, ICU admission, mortality, and in-hospital mortality) were developed using eXtreme Gradient Boosting (XGBoost). Medication and comorbidities were modelled separately, with both approaches incorporating demographic and socioeconomic features.

The dataset was split into a validation set (10%), and then outcomes were balanced by undersampling before being further divided into training (70%) and testing (30%) sets. The data partitioning process was uniformly applied across all datasets to maintain methodological consistency and model fairness. However, if there were underrepresented groups in the original data, they remained equally underrepresented in each partition. Categorical variables were one-hot encoded, and grid search hyperparameter tuning was applied to optimize the area under the receiver operating characteristic curve (AUROC) using log-loss. Models were trained using tenfold cross-validation, repeated 10 times. To enhance interpretability, SHapley Additive exPlanations (SHAP) were used to determine feature attributions [34]. SHAP summary plots were generated to assess feature importance and their effects on model predictions.

Finally, a sensitivity analysis was conducted on participants with arterial hypertension, diabetes mellitus, and lipid disorders to evaluate the impact of specific prescribed medications on the outcomes. Odds ratios (OR) with 95% confidence intervals (CI) were calculated. In all analyses, participants were stratified into two age groups: 18 to 65 and over 65.

Analyses were conducted using R software (version 4.3.0), with specific packages and code accessible in GitHub (https://github.com/bertamiro/XGBoost_code_COVID_outcomes).

## Results

### Characteristics of the cohort

**Fig. 1:**
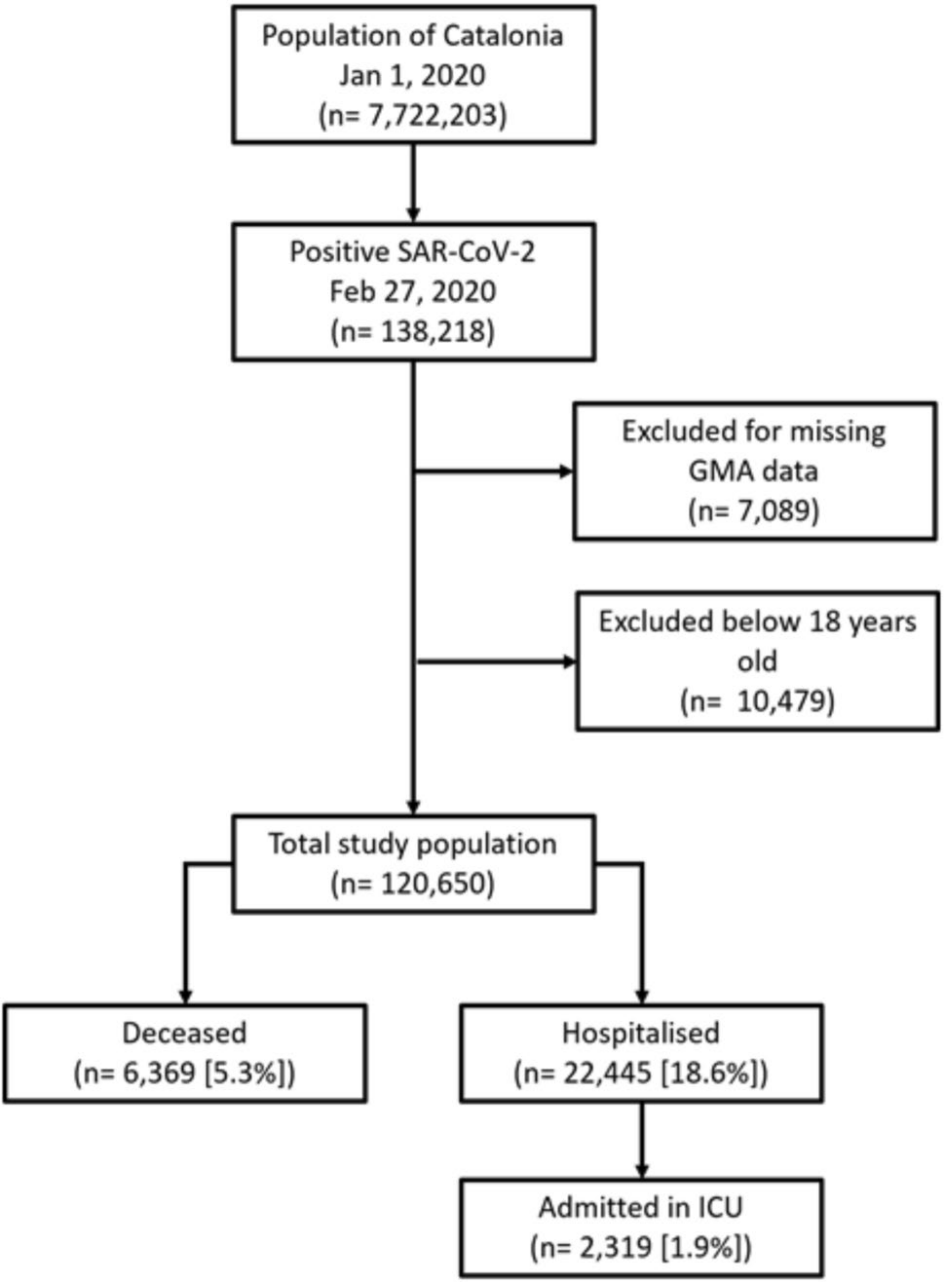
Description of the cohort used in the study. Data was collected from several data administrative databases centralized by AQuaS within the PADRIS framework. Those participants with missing GMA data were excluded from the study, as those younger than 18 years old. The final dataset used for analysis had 121,650 participants and included 255 variables spanning sociodemographic factors, comorbidities, chronic medication, and clinical outcomes

The final dataset had 121,650 participants (Fig. 1), with a mean age of 53 years (SD 20), 68,528 (57%) of which were female (Supplementary Material 2). In the logistic regression analysis for participants aged 18 to 65, age at exposure was the strongest predictor of hospitalisation (60–64 years OR 12.75, CI 11.03–14.82). Other factors were rituximab use (OR 15.19, CI 7.06–35.26), other antivirals (OR 14.37, CI 1.38–309.14), polypharmacy (OR 8.15, CI 4.06–16.52), and heparins (OR 8.07, CI 6.74–9.66). Among participants over 65, rituximab (OR 3.08 CI 1.56–6.31), calcineurin inhibitors (OR 3.01 CI 2.15–4.26), and Janus Kinase inhibitors (OR 2.96 CI 1.3–7.12) were most strongly associated with hospitalisation risk (Supplementary Material 3). Age was less important in the older groups (75–79 age OR 1.22, CI 1.13–1.31).

Risk of mortality among participants aged 18 to 65 was also determined by age (OR 74.21, CI 28.33–299.93). Other factors included rituximab use (OR 18.91, CI 4.49–54.1), polypharmacy (OR 16.3, CI 3.9–46.04), heart failure (OR 16.05, CI 11.35–22.09), and systemic corticosteroids (OR 15.39, CI 11.69–19.96) (Supplementary Material 4). In participants over 65, age remained a key determinant of mortality risk (85–89 years OR 4.15, CI 3.7–4.66), GMA score (very high risk GMA OR 2.87, CI 2.54–3.25), calcineurin inhibitors (OR 2.15, CI 1.48–3.07), and selective immunosuppressants (OR 1.97, CI 1.45–2.64; Supplementary Material 5).

The risk of ICU admission was also strongly associated with age (60–64 years 18.42, CI 11.82–30.71;), rituximab use (OR 14.87, CI 4.99–36.13), calcineurin inhibitors (OR 8.66, CI 5.51–13.02), direct-acting anticoagulants (OR 7.99, CI 4.57–13.03), and polypharmacy (OR 7.06, CI 1.69–19.9, Supplementary Material 6). Among participants over 65, the most relevant factors for ICU admission were calcineurin inhibitors (OR 3.53, CI 1.94–5.94), a history of transplants (OR 3.13, CI 1.72–5.24), male sex (OR 3.01, CI 2.65–3.43), and the use of selective immunosuppressants (OR 2.53, CI 1.46–4.1; Supplementary Material 7).

Finally, features associated with in-hospital death in participants aged 18 to 65 were age (60–64 years OR 8.53, CI 2.7–51.77), use of calcineurin inhibitors (OR 6.38, CI 3.72–10.39), selective immunosuppressants (OR 5.8, CI 3.49–9.2) or polypharmacy (OR 5.69, CI 1.31–17.51; Supplementary Material 8). Comparable characteristics to mortality risk, whether occurring in or outside the hospital were found relevant in participants over 65 (Supplementary Material 9).

### Machine learning model performance and interpretability

**Fig. 2:**
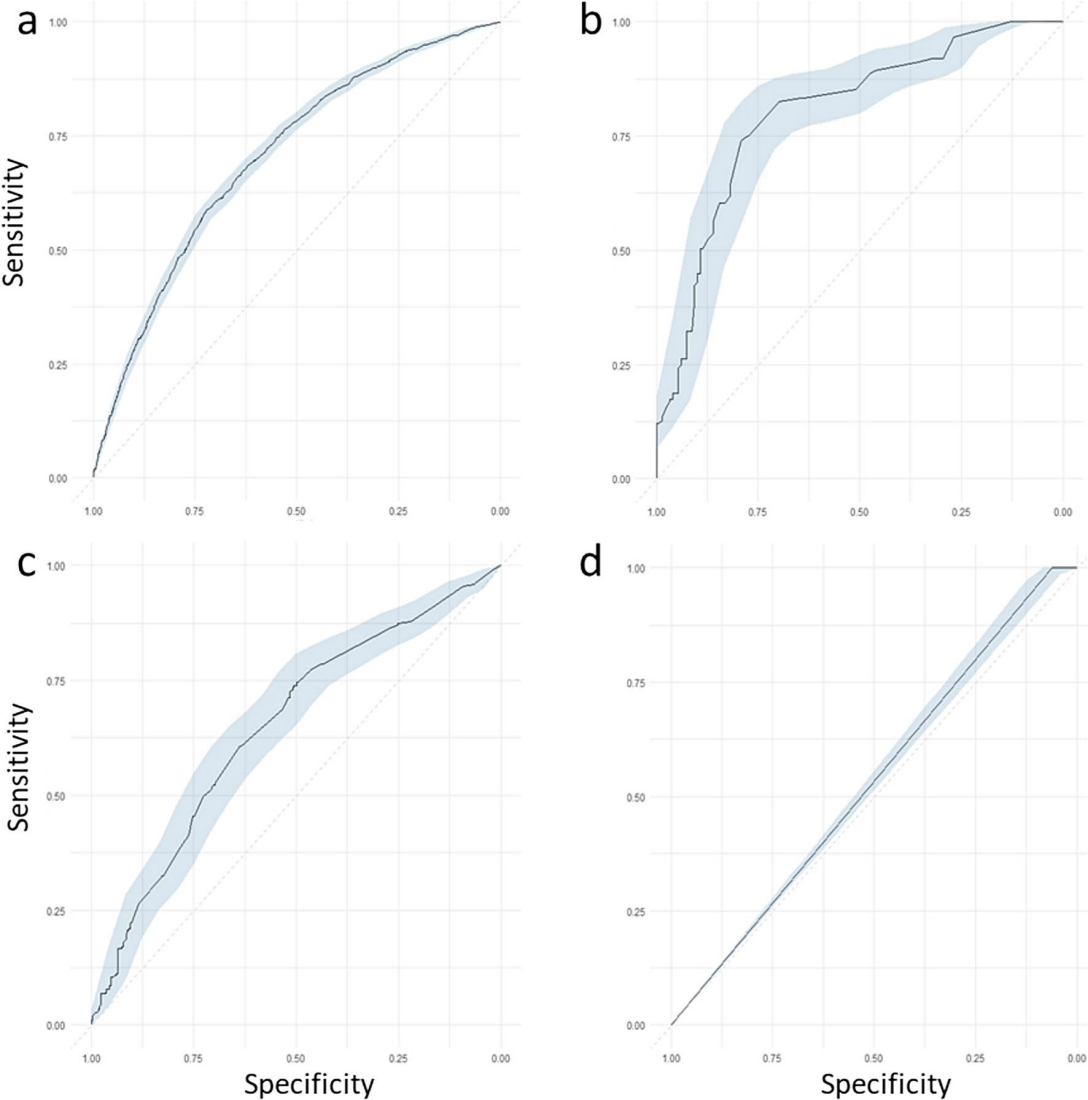
ROC values with a 95% CI for the pharmacological models evaluated in participants 18 to 65. XGBoost models for prescription medication-related features were built for the four outcomes: **a** risk of hospitalisation from COVID-19, **b** risk of death, **c** risk of ICU admission, and **d** risk of death while hospitalized separately in participants aged 18 to 65 years old

**Fig. 3:**
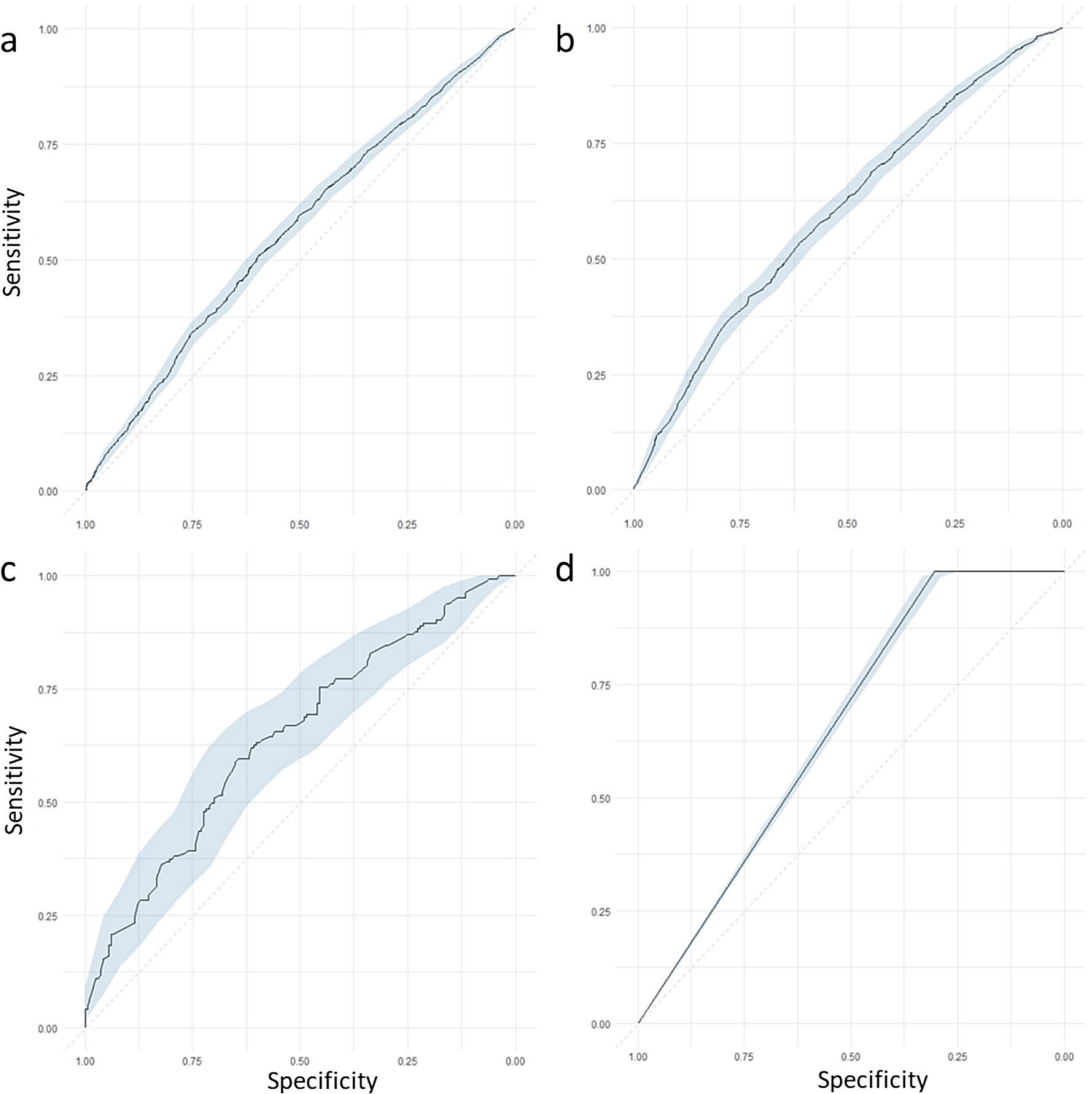
ROC values with a 95% CI for the pharmacological models evaluated in participants above 65. XGBoost models for prescription medication-related features were built for the four outcomes: **a** risk of hospitalisation from COVID-19, **b** risk of death, **c** risk of ICU admission, and **d** risk of death while hospitalized separately in participants aged older than 65 years

Eight distinct models were evaluated, one for each outcome and age group combinations. This approach allowed isolating the impact of pharmacological variables while minimizing collinearity with the disease-related variables for which these medications were prescribed. XGBoost was selected due to its robust tree-based ensemble, enabling the modelling of non-linear relationships, mitigation of predictor collinearity, and superior performance compared to K-Nearest Neighbors, AdaBoost, and Support Vector Machine methods (Supplementary Material 10).Pharmacological models demonstrated similar performance overall (Figs. 2 and 3). However, models for participants older than 65 performed worse. For hospitalisation risk, the model for participants aged 18 to 65 (Fig. 2a) achieved an AUC of 0.70 (CI 0.69–0.71), compared to 0.58 (CI 0.57–0.60) for those older than 65 (Fig. 3a). Similarly, for mortality risk in participants aged 18 to 65 (Fig. 2b) showed strong discrimination (AUC 0.89, CI 0.85–0.92), versus 0.65 (CI 0.63–0.67) model in older participants (Fig. 3b).

For ICU admission, the model for participants aged 18 to 65 (Fig. 2c) exhibited a similar performance to that of the model for older participants (Fig. 3c), with AUC values of 0.74 (CI 0.70–0.78) and 0.73 (CI 0.67–0.79), respectively. In contrast, the models predicting the risk of death during hospitalisation demonstrated greater variability: it achieved an AUC of 0.53 (CI 0.51–0.55) for participants aged 18 to 65, while it reached an AUC of 0.65 (CI 0.64–0.66) for those over 65 (Figs. 2d and 3d).

Models based on disease-related features performed similarly to those using medication data. The best performing model predicted risk of death in participants aged 18 to 65 (AUC 0.92, CI 0.89–0.95), while the hospitalisation model for those older than 65 showed the lowest performance (AUC 0.59, CI 0.58–0.61; Supplementary Material 11).

### Variable importance

**Fig. 4:**
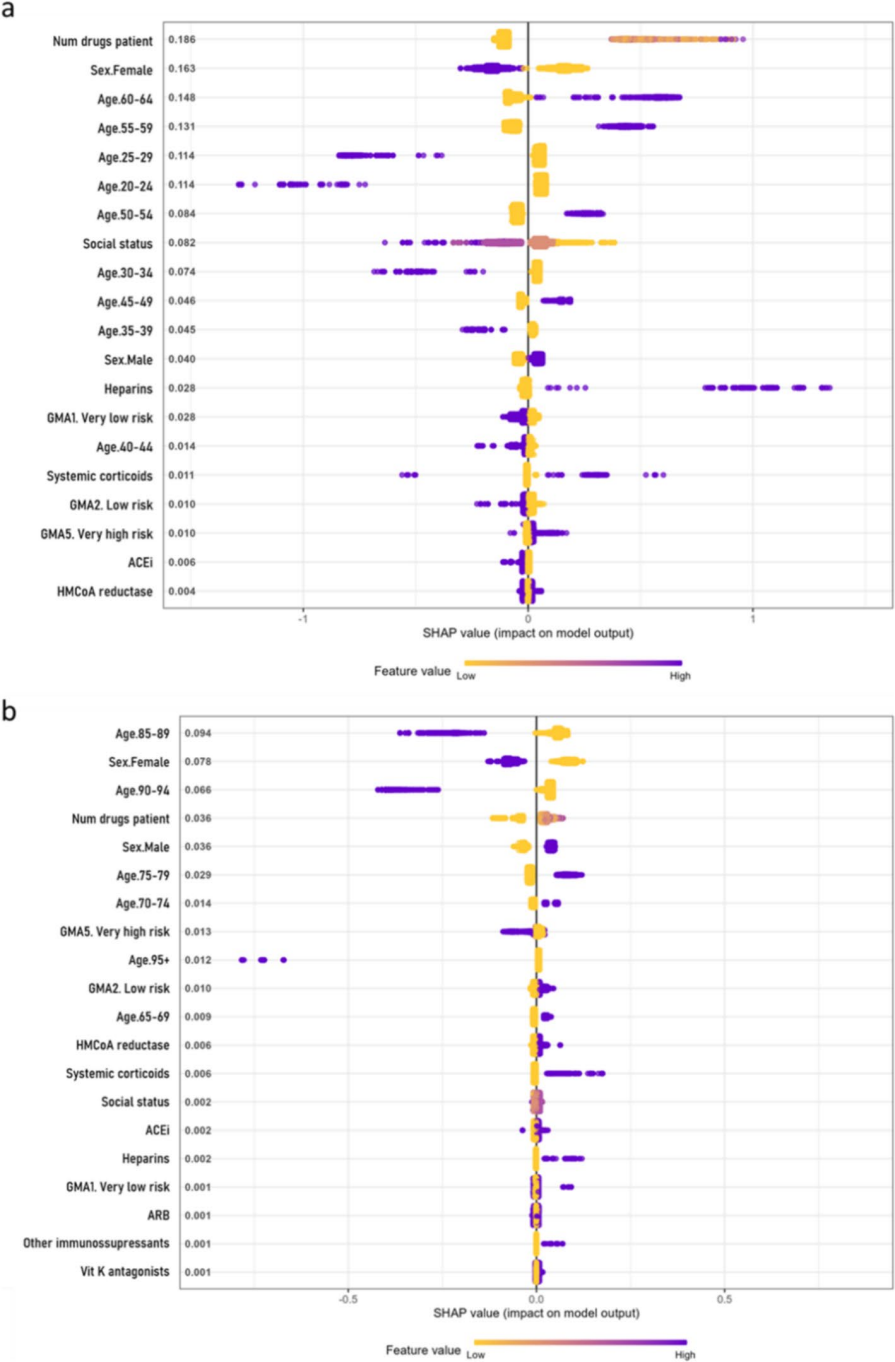
SHAP summary plot for hospitalisation risk in the chronic medication and demographics model. Variable importance (SHAP values) for the XGBoost model to determine the risk of hospitalisation from COVID-19 in participants by age groups: **a** from 18 to 65 years old, and **b** above 65 years old, for the pharmacological features model. SHAP values are ranked in descending order based on the absolute value of their influence on the XGBoost model; high values mean a higher probability of hospitalisation. Purple represents high variable values and yellow represents lower variable values (in the categorical variables one-hot-encoded, it is 0 yellow and 1 purple). Each point represents an instance (participant) for that variable in the dataset

Most models consistently identified age and sex as the most influential features, with older age and male sex linked to worst COVID-19 outcomes. In participants aged 18 to 65, hospitalisation risk was associated with the number of prescribed drugs and heparin use (Fig. 4a). In participants older than 65, the use of systemic corticosteroids was also linked to a higher risk (Fig. 4b).

**Fig. 5:**
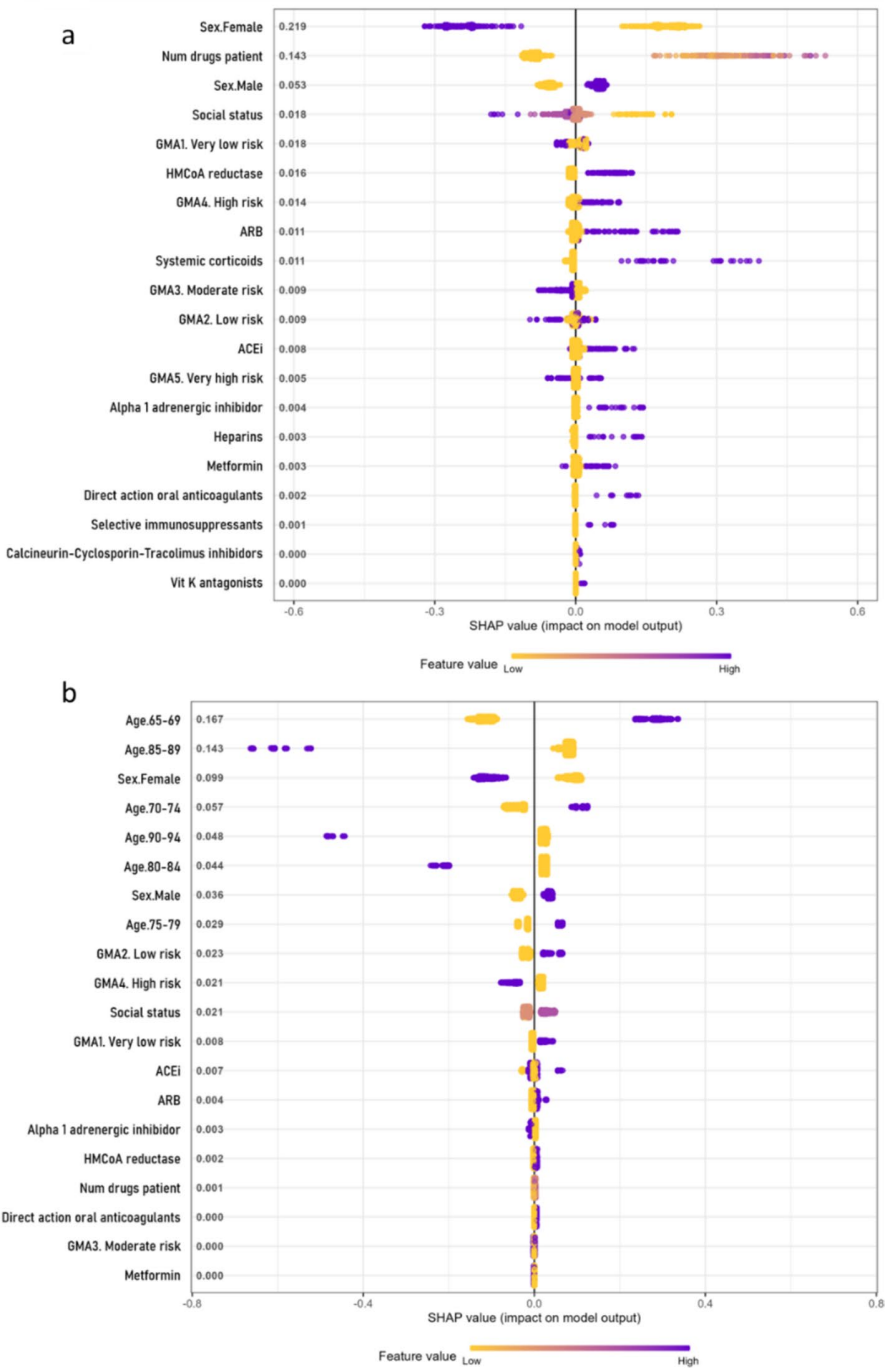
SHAP summary plot for mortality risk in the chronic medication and demographics model. Variable importance (SHAP values) for the XGBoost model to determine the risk of death from COVID-19 in participants by age groups: **a** from 18 to 65 years old, and **b** above 65 years old, for the pharmacological features model. SHAP values are ranked in descending order based on the absolute value of their influence on the XGBoost model; high values mean a higher probability of hospitalisation. Purple represents high variable values and yellow represents lower variable values (in the categorical variables one-hot-encoded, it is 0 yellow and 1 purple). Each point represents an instance (participant) for that variable in the dataset

In mortality models, key features for participants aged 18 to 65 included the use ACEi, ARBs, and HMG-CoA reductase inhibitors, all linked to increased risk (Fig. 5a); in those older than 65, ACEi and systemic corticosteroids were the main determinants (Fig. 5b).

**Fig. 6:**
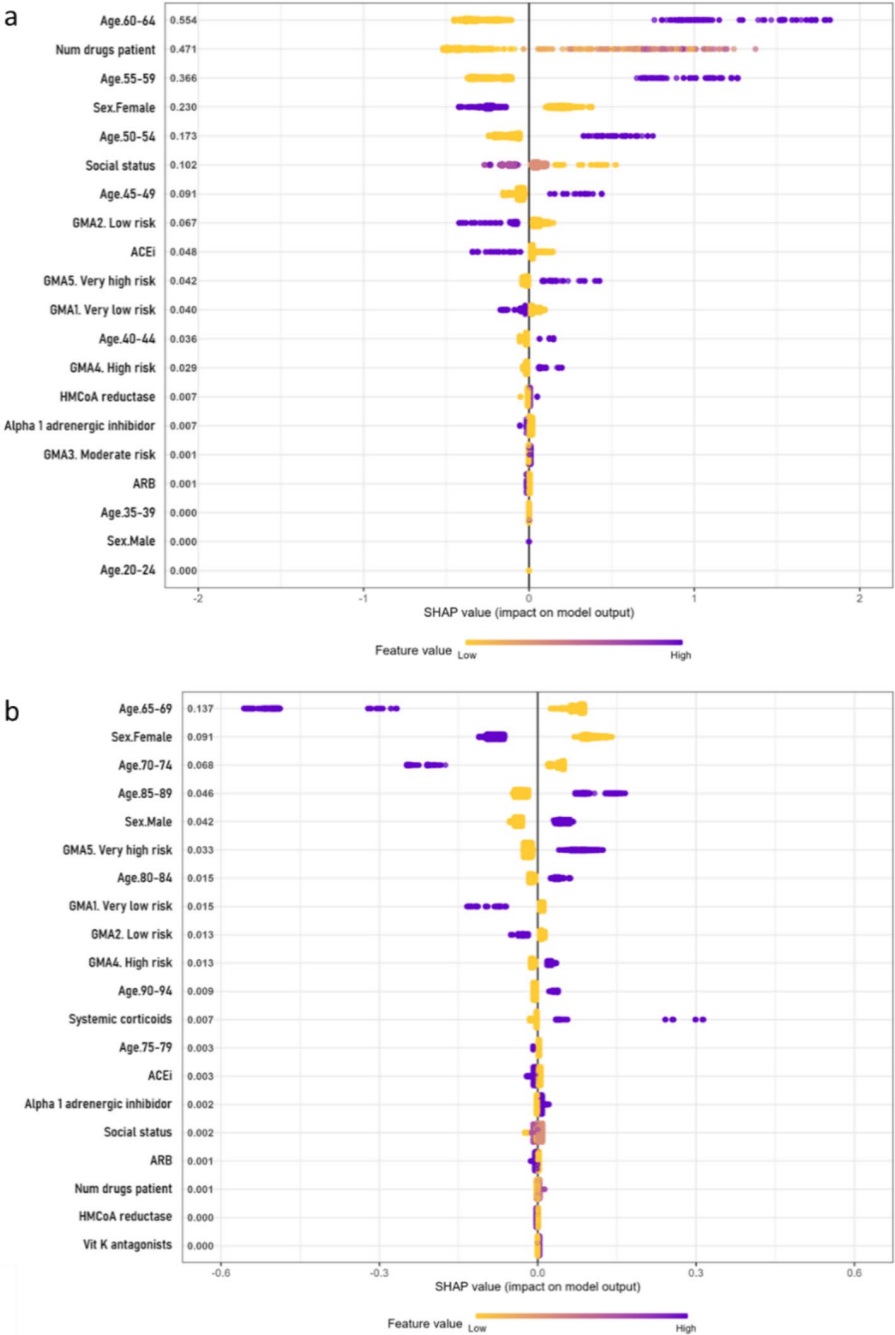
SHAP summary plot for ICU admission risk in the chronic medication and demographics model. Variable importance (SHAP values) for the XGBoost model to determine the risk of ICU admission from COVID-19 in participants by age groups: **a** from 18 to 65 years old, and **b** above 65 years old, for the pharmacological features model. SHAP values are ranked in descending order based on the absolute value of their influence on the XGBoost model; high values mean a higher probability of hospitalisation. Purple represents high variable values and yellow represents lower variable values (in the categorical variables one-hot-encoded, it is 0 yellow and 1 purple). Each point represents an instance (participant) for that variable in the dataset

Predictors of ICU admission in both age groups included the use of HMG-CoA reductase inhibitors and ACEi or ARBs (Fig. 6a, b). While ACEi and ARBs were linked to a slightly lower ICU admission risk, participants using HMG-CoA reductase inhibitors were at higher risk.

In models evaluating COVID-19 outcomes and chronic diseases, main predictors of hospitalisation in participants aged 18 to 65 included age, sex, obesity, social status, smoking, and metabolic disorders (Supplementary Material 12), while in those older than 65, key features were age, dementia, sex, and social status (Supplementary Material 12). Mortality risk models identified age, sex, neoplasms, social status, COPD, and diabetes as significant predictors in the 18 to 65 age group (Supplementary Material 13); whereas age, sex, renal insufficiency, and metabolic disorders were the most relevant factors in those older than 65 (Supplementary Material 13).

Finally, the top predictors of ICU admission in participants aged 18 to 65 were sex, hypertension, obesity, and diabetes (Supplementary Material 14), whereas in those older than 65, age, sex, dementia, metabolic disorders, and GMA were most relevant (Supplementary Material 14). Local model predictions for hospitalisation risk in three representative participants aged 18 to 65 are shown in Supplementary Material 15, with accurate predictions at p 0.05.

### Sensitivity analysis

A multivariate analysis was conducted to evaluate the relationship between selected comorbidities and their associated medications, specifically hypertension (ACEi and ARBs), diabetes (biguanides and DPP4 inhibitors), and metabolic disorders (statins).

Participants receiving ACEi or ARBs for hypertension did not show an increased risk for any of the outcomes compared to those with compared to patients not using ACEi or ARBs to treat hypertension.. However, in individuals over 65, treatment with ACEi and ARBs was associated with a reduced risk of death (OR 0.78, 95% CI 0.68–0.92 and OR 0.8, 95% CI: 0.68–0.95, respectively, Supplementary Material 17). Among participants aged 65 or below, none of the analysed medications were linked to an increased risk of hospitalisation or ICU admission (Supplementary Material 16, Supplementary Material 18).

For diabetic participants, treatment with DPP4 inhibitors in the 18 to 65 age group was associated with a higher risk of death (OR 3.03, 95% CI 1.17–7.00, Supplementary Material 17). In contrast, among participants over 65, biguanides demonstrated a protective effect on overall mortality (OR 0.79, 95% CI 0.68–0.92), an effect not observed with DPP4 inhibitors (OR 1.2, 95% CI 0.96–1.49). Furthermore, when comparing biguanides to DPP4 inhibitors in participants older than 65, biguanides consistently showed better outcomes across all measures. Statins did not provide a protective effect for any of the assessed outcomes in either age group.

## Discussion

### Statement of key findings

This study showed that models for chronic medication exhibited AUCROC values ranging from 0.70 to 0.89 for COVID-19 participants aged 18 to 65; however, these predictions showed a poorer fit in participants older than 65 for all tested outcomes (AUROC between 0.58 and 0.73). This could be attributed to the more complex context observed in elderly participants. Age, sex, number of drugs, and previous pathologies were consistently important predictors across models. Hypertension drugs such as ACEi and ARBs were associated especially in the risk of death and ICU admission in participants aged 18 to 65. By contrast, the sensitivity study found that in participants with arterial hypertension older than 65, ACEi and ARBs were protective factors for death when compared with participants with arterial hypertension without other medications (OR 0.78, and OR 0.8, respectively).

### Interpretation

We conducted a retrospective study of chronic medication use employing XGBoost to identify key factors and predict relevant outcomes. The models achieved robust predictive power for COVID-19 participants aged 18 to 65, but showed diminished performance in participants over 65, likely reflecting population heterogeneity and unaccounted confounding factors [35]. While prior research examined chronic medication, many studies had smaller sample sizes or relied on population-based statistical methods [36]. This study highlighted the role of age, sex, chronic medication, socioeconomic factors, and pre-existing conditions in predicting COVID-19 outcomes. Our findings align with previous evidence identifying these factors and pre-existing comorbidities as key determinants of COVID-19 severity [37–41].

As pathologies increase with age, so does medication use and its associated side effects, all of which influence COVID-19 severity [42]. We found that the number of prescribed drugs correlated with a higher risk of negative outcomes. Other studies have linked polypharmacy to increased vulnerability [27, 43] and a higher risk of hospitalisation and mortality from COVID-19.

Conflicting findings regarding ACEi and ARBs emphasise the need for subpopulation-specific analyses [44–46]. Although these drugs were associated with a higher risk of death when compared with participants who did not take antihypertensive drugs, they were inversely associated with ICU admission risk in the same age group. While XGBoost suggested an association between these drugs and increased mortality in participants aged 18 to 65, our sensitivity analysis did not confirm this. Instead, we found a protective effect in hypertensive participants taking these drugs compared with hypertensive participants taking other antihypertensive drugs [47]. The discrepancy likely arises from differences in cohort composition; XGBoost included both hypertensive and non-hypertensive participants, but the sensitivity analysis focused solely on hypertensive individuals.

We also observed a potential protective effect of metformin against severe COVID-19 outcomes, likely due to its immunomodulatory properties [48]. This aligns with hypotheses suggesting its role in mitigating hyper-inflammatory responses. In the sensitivity study, treatment with DPP4 inhibitors conferred higher probability of death in younger diabetic participants [49], whereas biguanides had a protective effect on overall fatality rates in diabetic participants over 65 [50]. Metformin could reduce viral affinity for angiotensin-converting enzyme 2 and inhibit inflammation via adenosine monophosphate-activated protein activation, potentially protecting against severe COVID-19 [51, 52].

ML-driven patient prioritization tools are needed in emergency settings [53]; our analysis of medications associated with severe COVID-19 outcomes represents a foundational step toward their development.

Our results highlight strong associations between socioeconomic vulnerability and COVID-19 severity and mortality [54, 55].

### Strengths and weaknesses

This study uniquely analyses the relationship between prescribed chronic medication and COVID-19 outcomes using a large cohort. Our findings are concordant with other populations studied with different methodologies, reinforcing their robustness. Sensitivity analyses further provided insights into specific medications within hypertensive and diabetic subpopulations.

However, the study has limitations. Results depend on age and sex, factors that may limit the impact of other predictors. Additionally, medication data, sourced from community and hospital pharmacy invoicing systems, cannot guarantee adherence. To mitigate this, only medications dispensed within three months prior to inclusion were considered. Moreover, medications were grouped by active ingredient, without accounting for dosage variations. Furthermore, the evolving COVID-19 management criteria during the pandemic introduced potential inconsistencies. Lastly, models performed poorly for participants over 65, limiting results interpretation for this relevant age group.

### Future research

Future studies should investigate the mechanisms underlying the protective effects of ACEi, ARBs, and metformin in specific subpopulations. Additionally, research to develop predictive models tailored to elderly participants is still needed, since they are more prone to severe COVID-19 outcomes. It would be interesting to conduct prospective studies to validate these findings and explore causal relationships between chronic medication and COVID-19 outcomes. Finally, the impact of socioeconomic factors on COVID-19 outcomes requires further exploration to design equitable healthcare interventions.

## Conclusion

Machine learning predictive models have high performance predicting hospitalisation, ICU admission, and death in COVID-19 participants. Key factors influencing outcomes include age, sex, comorbidities, socioeconomic status and chronic medication. Our findings support decreased risks of poor outcomes in participants with arterial hypertension under ACEi or ARBs treatment. Additionally, biguanides showed potential protective effects for participants with diabetes. Participants taking metformin, ACEi, or ARBs should not be switched to alternative medications in relation to COVID-19.

Our findings leveraged administrative databases and advanced AI querying tools. Given their regular updates and compliance with EU data protection regulations, the methodology is readily applicable with limited additional system cost.

## Supplementary Information

Below is the link to the electronic supplementary material.

Supplementary file1 (DOCX 1619 kb)
