# Supplementary material for: A machine learning model exploring the relationship between chronic medication and COVID-19 clinical outcomes
Source: Int J Clin Pharm. 2025 Jul 28;47(4):1075–86. doi: 10.1007/s11096-025-01955-7 (PMC12335402; doi:10.1007/s11096-025-01955-7)
Supplement: Supplementary file 1 — Supplementary file1 (DOCX 1619 kb) [file 11096_2025_1955_MOESM1_ESM.docx]

# Title: A machine learning model exploring the relationship between chronic medication and COVID-19 clinical outcomes

International Journal of Clinical Pharmacy

**Supplementary Material 1. Full list of drugs included in the study.** The drugs were grouped by active ingredient. The first level was used to build the models.

- Other immunosuppressants
  - Methotrexate
  - Azathioprine
- Calcineurin inhibitors
  - Tacrolimus
  - Cyclosporine
- Tumor necrosis factor (TNF)-alpha inhibitors
  - Etanercept
  - Infliximab
  - Adalimumab
  - Certolizumab
  - Certolizumab pegol
  - Golimumab
- Rituximab
- Janus kinase inhibitors
  - Ruxolitinib
  - Baricitinib
  - Tofacitinib
- Interleukin inhibitors
  - Anakinra
  - Tocilizumab
  - Sarilumab
  - Ustekinumab
- Selective immunosuppressants
  - Natalizumab
  - Leflunomide
  - Eculizumab
  - Abatacept
  - Mycophenolic acid
  - Sirolimus
- Cox-2 inhibitors
  - Celecoxib
  - Etoricoxib
- Systemic corticosteroids
  - Prednisone
  - Prednisolone
  - Dexamethasone
  - Deflazacort
  - Metilprednisolone
  - Hydrocortisone
  - Fludrocortisone
- Biguanides
  - Metformin
- Dipeptidyl peptidase 4 (DPP-4) inhibitors
  - Sitagliptin
  - Linagliptin
  - Alogliptin
  - Vildagliptin
  - Saxagliptin
- Oral anticoagulants
  - Rivaroxaban
  - Edoxaban
  - Apixaban
  - Dabigatran etexilate
- Vitamin K antagonists
  - Warfarin
  - Acenocumarol
- Heparines
  - Enoxaparin
  - Bemiparin
  - Tinzaparin
  - Dalteparin
  - Nadroparin
- Alpha-1 adrenergic receptor antagonists
  - Doxazosin
  - Tamsulosin
  - Tamsulosin and dutasteride
  - Tamsulosin and solifenacin
  - Silodosin
  - Terazosin
  - Alfuzosin
  - Prazosin
- Angiotensin converting enzyme inhibitors (ACEi) and combinations
  - Enalapril and diuretics
  - Enalapril
  - Perindopril
  - Captopril
  - Lisinopril and diuretics
  - Fosinopril
  - Lisinopril
  - Ramipril
  - Ramipril and diuretics
  - Perindopril and diuretics
  - Imidapril
  - Trandolapril and diuretics
  - Captopril and diuretics
  - Quinapril and diuretics
  - Delapril and diuretics
  - Cilazapril and diuretics
  - Quinapril
  - Trandolapril
  - Enalapril and lercanidipine
  - Perindopril and amlodipine
  - Fosinopril and diuretics
  - Enalapril and nitrendipine
- Angiotensin receptor blockers (ARBs or angiotensin II inhibitors) and combinations
  - Losartan
  - Candesartan
  - Losartan and diuretics
  - Irbesartan
  - Telmisartan
  - Valsartan and diuretics
  - Valsartan and amlodipine
  - Valsartan and sacubitril
  - Telmisartan and diuretics
  - Valsartan, amlodipine and hydrochlorothiazide
  - Olmesartan medoxomil
  - Valsartan
  - Olmesartan medoxomil and diuretics
  - Olmesartan medoxomil and amlodipine
  - Olmesartan medoxomil, amlodipine and hydrochlorothiazide
  - Candesartan and diuretics
  - Irbesartan and diuretics
  - Eprosartan and diuretics
  - Telmisartan and amlodipine
  - Eprosartan
- Hydroxymethylglutaryl-CoA (HMG-CoA) reductase inhibitors or statins
  - Atorvastatine
  - Simvastatine
  - Rosuvastatin
  - Pitavastatin
  - Pravastatin
  - Fluvastatin
  - Lovastatin
- Antimalarial drugs
  - Chloroquine
  - Hydroxychloroquine
- Protease inhibitors
  - Darunavir and cobicistat
  - Darunavir
  - Ritonavir
  - Atazanavir and cobicistat
- Nucleoside and nucleotide reverse transcriptase inhibitors
  - Lamivudine
  - Tenofovir disoproxil
  - Abacavir
  - Lamivudine and abacavir
  - Lamivudine and abacavir
  - Emtricitabine and tenofovir alafenamide
  - Emtricitabine, tenofovir alafenamide and rilpivirine
  - Emtricitabine, tenofovir disoproxil and efavirenz
  - Emtricitabine, tenofovir_disoproxil and rilpivirine
  - Tenofovir disoproxil and emtricitabine
  - Entecavir
- Non-nucleoside reverse transcriptase inhibitors
  - Etravirine
  - Efavirenz
  - Nevirapine
  - Rilpivirine
- Integrase inhibitors
  - Dolutegravir
  - Raltegravir
  - Emtricitabine, tenofovir alafenamide, elvitegravir and cobicistat
  - Emtricitabine, tenofovir alafenamide, darunavir and cobicistat
  - Lamivudine, abacavir and dolutegravir
  - Emtricitabine, tenofovir alafenamide and bictegravir
  - Dolutegravir and rilpivirine
  - Emtricitabine, tenofovir disoproxil, elvitegravir and cobicistat
  - Lamivudine and dolutegravir
- Other antivirals
  - Maraviroc
  - Enfuvirtide

**Supplementary Material 2. Socio-demographic and health characteristics of hospitalised participants aged 18 to 65 in the cohort studied.** Odds Ratio (OR) estimates and 95% confidence intervals (CI) for significant socio-demographic, chronic illness, and medication variables in univariate logistic regression for the risk of hospitalisation in participants diagnosed with COVID-19. Data are presented as the total number of participants and the percentage (%) in relation to the overall cohort for categorical variables, and mean and standard deviation (SD) for numerical variables, using a univariate model by age groups (18 to 65 years old). Probabilities were calculated using the Student’s t-test for continuous variables and chi-squared for categorical variables (N = 85,994). The reference group for disease and drug variables was “No”.

| **Variable** | **Levels** | **Not hospitalised 75483 (87.78%)** | **Hospitalised 10511 (12.22%)** | **OR (CI)** | **Pr(>\|z\|)** | **Adjusted-p-val BY** |
| --- | --- | --- | --- | --- | --- | --- |
| Sex | Female | 43539 (57.7%) | 4462 (42.5%) | 1 | (reference level) | (reference level) |
|  | Male | 31944 (42.3%) | 6049 (57.5%) | 1.85 (1.77-1.93) | <0.001 | <0.001 |
| Age at exposure | 20-24 | 7252 (9.6%) | 204 (1.9%) | 1 | (reference level) | (reference level) |
|  | 25-29 | 8081 (10.7%) | 337 (3.2%) | 1.48 (1.24-1.77) | <0.001 | <0.001 |
|  | 30-34 | 7616 (10.1%) | 447 (4.3%) | 2.09 (1.77-2.47) | <0.001 | <0.001 |
|  | 35-39 | 8976 (11.9%) | 729 (6.9%) | 2.89 (2.47-3.39) | <0.001 | <0.001 |
|  | 40-44 | 10353 (13.7%) | 1118 (10.6%) | 3.84 (3.3-4.48) | <0.001 | <0.001 |
|  | 45-49 | 10002 (13.3%) | 1506 (14.3%) | 5.35 (4.62-6.23) | <0.001 | <0.001 |
|  | 50-54 | 9151 (12.1%) | 1856 (17.7%) | 7.21 (6.24-8.38) | <0.001 | <0.001 |
|  | 55-59 | 7962 (10.5%) | 2130 (20.3%) | 9.51 (8.23-11.05) | <0.001 | <0.001 |
|  | 60-64 | 6090 (8.1%) | 2184 (20.8%) | 12.75 (11.03-14.82) | <0.001 | <0.001 |
| Social status | 1 | 2451 (3.2%) | 574 (5.5%) | 1 | (reference level) | (reference level) |
|  | 2 | 45814 (60.7%) | 6078 (57.8%) | 0.57 (0.52-0.62) | <0.001 | <0.001 |
|  | 3 | 26456 (35%) | 3756 (35.7%) | 0.61 (0.55-0.67) | <0.001 | <0.001 |
|  | 4 | 762 (1%) | 103 (1%) | 0.58 (0.46-0.72) | <0.001 | <0.001 |
| GMA Cat | 1. Very low risk | 31494 (41.7%) | 3503 (33.3%) | 1 | (reference level) | (reference level) |
|  | 2. Low risk | 19690 (26.1%) | 2913 (27.7%) | 1.33 (1.26-1.4) | <0.001 | <0.001 |
|  | 3. Moderate risk | 11162 (14.8%) | 1783 (17%) | 1.44 (1.35-1.53) | <0.001 | <0.001 |
|  | 4. High risk | 8617 (11.4%) | 1461 (13.9%) | 1.52 (1.43-1.63) | <0.001 | <0.001 |
|  | 5. Very high risk | 4520 (6%) | 851 (8.1%) | 1.69 (1.56-1.83) | <0.001 | <0.001 |
| Diabetes | 0 | 72299 (95.8%) | 9219 (87.7%) | 1 | (reference level) | (reference level) |
|  | 1 | 3184 (4.2%) | 1292 (12.3%) | 3.18 (2.97-3.41) | <0.001 | <0.001 |
| Hypertension | 0 | 68941 (91.3%) | 8396 (79.9%) | 1 | (reference level) | (reference level) |
|  | 1 | 6542 (8.7%) | 2115 (20.1%) | 2.65 (2.51-2.8) | <0.001 | <0.001 |
| Neoplasm | 0 | 72982 (96.7%) | 9724 (92.5%) | 1 | (reference level) | (reference level) |
|  | 1 | 2501 (3.3%) | 787 (7.5%) | 2.36 (2.17-2.56) | <0.001 | <0.001 |
| Asthma | 0 | 71318 (94.5%) | 9826 (93.5%) | 1 | (reference level) | (reference level) |
|  | 1 | 4165 (5.5%) | 685 (6.5%) | 1.19 (1.1-1.3) | <0.001 | <0.001 |
| Dementia | 0 | 75280 (99.7%) | 10417 (99.1%) | 1 | (reference level) | (reference level) |
|  | 1 | 203 (0.3%) | 94 (0.9%) | 3.35 (2.61-4.26) | <0.001 | <0.001 |
| Ictus | 0 | 74495 (98.7%) | 10148 (96.5%) | 1 | (reference level) | (reference level) |
|  | 1 | 988 (1.3%) | 363 (3.5%) | 2.7 (2.38-3.04) | <0.001 | <0.001 |
| Heart Failure | 0 | 75210 (99.6%) | 10312 (98.1%) | 1 | (reference level) | (reference level) |
|  | 1 | 273 (0.4%) | 199 (1.9%) | 5.32 (4.42-6.38) | <0.001 | <0.001 |
| Renal Insufficiency | 0 | 74996 (99.4%) | 10219 (97.2%) | 1 | (reference level) | (reference level) |
|  | 1 | 487 (0.6%) | 292 (2.8%) | 4.4 (3.8-5.09) | <0.001 | <0.001 |
| Ischemia | 0 | 74680 (98.9%) | 10098 (96.1%) | 1 | (reference level) | (reference level) |
|  | 1 | 803 (1.1%) | 413 (3.9%) | 3.8 (3.37-4.29) | <0.001 | <0.001 |
| COPD | 0 | 74545 (98.8%) | 10139 (96.5%) | 1 | (reference level) | (reference level) |
|  | 1 | 938 (1.2%) | 372 (3.5%) | 2.92 (2.58-3.29) | <0.001 | <0.001 |
| Hematological Neoplasm | 0 | 75314 (99.8%) | 10448 (99.4%) | 1 | (reference level) | (reference level) |
|  | 1 | 169 (0.2%) | 63 (0.6%) | 2.69 (2-3.57) | <0.001 | <0.001 |
| Obesity | 0 | 65947 (87.4%) | 8003 (76.1%) | 1 | (reference level) | (reference level) |
|  | 1 | 9536 (12.6%) | 2508 (23.9%) | 2.17 (2.06-2.28) | <0.001 | <0.001 |
| Transplants | 0 | 75387 (99.9%) | 10425 (99.2%) | 1 | (reference level) | (reference level) |
|  | 1 | 96 (0.1%) | 86 (0.8%) | 6.48 (4.83-8.67) | <0.001 | <0.001 |
| Metabolic Syndrome | 0 | 68349 (90.5%) | 8486 (80.7%) | 1 | (reference level) | (reference level) |
|  | 1 | 7134 (9.5%) | 2025 (19.3%) | 2.29 (2.17-2.41) | <0.001 | <0.001 |
| HIV | 0 | 74916 (99.2%) | 10397 (98.9%) | 1 | (reference level) | (reference level) |
|  | 1 | 567 (0.8%) | 114 (1.1%) | 1.45 (1.18-1.77) | <0.001 | <0.001 |
| Other Immunosuppressants | 0 | 75202 (99.6%) | 10441 (99.3%) | 1 | (reference level) | (reference level) |
|  | 1 | 281 (0.4%) | 70 (0.7%) | 1.79 (1.37-2.32) | <0.001 | <0.001 |
| Inhib Calcineurin | 0 | 75368 (99.8%) | 10410 (99%) | 1 | (reference level) | (reference level) |
|  | 1 | 115 (0.2%) | 101 (1%) | 6.36 (4.86-8.31) | <0.001 | <0.001 |
| Anti TNF | 0 | 75335 (99.8%) | 10476 (99.7%) | 1 | (reference level) | (reference level) |
|  | 1 | 148 (0.2%) | 35 (0.3%) | 1.7 (1.16-2.43) | 0.005 | 0.01 |
| Rituximab | 0 | 75474 (100%) | 10492 (99.8%) | 1 | (reference level) | (reference level) |
|  | 1 | 9 (0%) | 19 (0.2%) | 15.19 (7.06-35.26) | <0.001 | <0.001 |
| Selective Immunosuppressants | 0 | 75316 (99.8%) | 10390 (98.8%) | 1 | (reference level) | (reference level) |
|  | 1 | 167 (0.2%) | 121 (1.2%) | 5.25 (4.15-6.63) | <0.001 | <0.001 |
| Systemic Corticosteroids | 0 | 74980 (99.3%) | 10190 (96.9%) | 1 | (reference level) | (reference level) |
|  | 1 | 503 (0.7%) | 321 (3.1%) | 4.7 (4.07-5.41) | <0.001 | <0.001 |
| Metformin | 0 | 74361 (98.5%) | 10059 (95.7%) | 1 | (reference level) | (reference level) |
|  | 1 | 1122 (1.5%) | 452 (4.3%) | 2.98 (2.66-3.33) | <0.001 | <0.001 |
| Inhib DPP4 | 0 | 75311 (99.8%) | 10441 (99.3%) | 1 | (reference level) | (reference level) |
|  | 1 | 172 (0.2%) | 70 (0.7%) | 2.94 (2.21-3.86) | <0.001 | <0.001 |
| Direct Action Oral Anticoagulants | 0 | 75385 (99.9%) | 10455 (99.5%) | 1 | (reference level) | (reference level) |
|  | 1 | 98 (0.1%) | 56 (0.5%) | 4.12 (2.95-5.7) | <0.001 | <0.001 |
| VitK Antagonists | 0 | 75326 (99.8%) | 10428 (99.2%) | 1 | (reference level) | (reference level) |
|  | 1 | 157 (0.2%) | 83 (0.8%) | 3.82 (2.91-4.97) | <0.001 | <0.001 |
| Heparins | 0 | 75253 (99.7%) | 10258 (97.6%) | 1 | (reference level) | (reference level) |
|  | 1 | 230 (0.3%) | 253 (2.4%) | 8.07 (6.74-9.66) | <0.001 | <0.001 |
| Alpha1 adrenergic inhib | 0 | 75007 (99.4%) | 10248 (97.5%) | 1 | (reference level) | (reference level) |
|  | 1 | 476 (0.6%) | 263 (2.5%) | 4.04 (3.47-4.7) | <0.001 | <0.001 |
| ACEi combinations | 0 | 72609 (96.2%) | 9535 (90.7%) | 1 | (reference level) | (reference level) |
|  | 1 | 2874 (3.8%) | 976 (9.3%) | 2.59 (2.4-2.79) | <0.001 | <0.001 |
| ARB combinations | 0 | 73883 (97.9%) | 9939 (94.6%) | 1 | (reference level) | (reference level) |
|  | 1 | 1600 (2.1%) | 572 (5.4%) | 2.66 (2.41-2.93) | <0.001 | <0.001 |
| HMCoA reductase | 0 | 72871 (96.5%) | 9500 (90.4%) | 1 | (reference level) | (reference level) |
|  | 1 | 2612 (3.5%) | 1011 (9.6%) | 2.97 (2.75-3.2) | <0.001 | <0.001 |
| Antimalarials | 0 | 75371 (99.9%) | 10481 (99.7%) | 1 | (reference level) | (reference level) |
|  | 1 | 112 (0.1%) | 30 (0.3%) | 1.93 (1.26-2.84) | 0.001 | <0.001 |
| Inhib TI nucleosides and nucleotides | 0 | 75345 (99.8%) | 10468 (99.6%) | 1 | (reference level) | (reference level) |
|  | 1 | 138 (0.2%) | 43 (0.4%) | 2.24 (1.57-3.13) | <0.001 | <0.001 |
| Inhib Integrase | 0 | 75210 (99.6%) | 10459 (99.5%) | 1 | (reference level) | (reference level) |
|  | 1 | 273 (0.4%) | 52 (0.5%) | 1.37 (1.01-1.83) | 0.038 | 0.05 |
| Other Antivirals | 0 | 75482 (100%) | 10509 (100%) | 1 | (reference level) | (reference level) |
|  | 1 | 1 (0%) | 2 (0%) | 14.37 (1.38-309.14) | 0.03 | 0.05 |
| Num drugs patient |  | 0.16 (0.56) | 0.48 (0.95) | 1.72 (1.68-1.76) | <0.001 | <0.001 |
| Polypharmacy |  | 0.0002 (0.014) | 0.0016 (0.04) | 8.15 (4.06-16.52) | <0.001 | <0.001 |
| GMA: Adjusted morbidity groups. Social status: 1) exempted (non-working population or non-contributory pension recipients); 2) < 18,000€ income per year; 3) 18,000€ to 100,000€ income per year; and 4) > 100,000€ per year. ACEi: Angiotensin-converting-enzyme inhibitors, ARB: Angiotensin receptor blockers, DPP4: Dipeptidyl peptidase 4, HIV: human immunodeficiency virus, HMCoA: ß-Hydroxy ß-methylglutaryl-CoA, Inhib: inhibitors, COPD: Chronic obstructive pulmonary disease, TI: reverse transcriptase, TNF: Tumor necrosis factor, Vit K: vitamine K. The adjusted p-value was computed using the Benjamini-Yekutieli (BY) correction method. | | | | | | |

**Supplementary Material 3. Socio-demographic and health characteristics of hospitalised participants over 65 years in the cohort studied.** Odds Ratio (OR) estimates and 95% confidence intervals (CI) for significant socio-demographic, chronic illness, and medication variables in univariate logistic regression for the risk of hospitalisation in participants diagnosed with COVID-19. Data are presented as the total number of participants and the percentage (%) in relation to the overall cohort for categorical variables, and mean and standard deviation (SD) for numerical variables, using a univariate model by age groups (18 to 65 years old). Probabilities were calculated using the Student’s t-test for continuous variables and chi-squared for categorical variables (N = 34,656). The reference group for disease and drug variables was “No”.

| **Variable** | **Levels** | **Not hospitalised 22722 (65.56%)** | **Hospitalised 11934 (34.44%)** | **OR (CI)** | **Pr(>\|z\|)** | **Adjusted-p-val BY** |
| --- | --- | --- | --- | --- | --- | --- |
| Sex | Female | 14745 (64.9%) | 5782 (48.4%) | 1.97 (1.88-2.06) | (reference level) | (reference level) |
|  | Male | 7977 (35.1%) | 6152 (51.6%) |  | <0.001 | <0.001 |
| Age at exposure | 65-69 | 3792 (16.7%) | 2150 (18%) |  | (reference level) | (reference level) |
|  | 70-74 | 3531 (15.5%) | 2382 (20%) | 1.19 (1.1-1.28) | <0.001 | <0.001 |
|  | 75-79 | 3555 (15.6%) | 2449 (20.5%) | 1.22 (1.13-1.31) | <0.001 | <0.001 |
|  | 80-84 | 3883 (17.1%) | 2128 (17.8%) | 0.97 (0.9-1.04) | 0.373 | 0.457 |
|  | 85-89 | 4937 (21.7%) | 2016 (16.9%) | 0.72 (0.67-0.78) | <0.001 | <0.001 |
|  | 90-94 | 2750 (12.1%) | 763 (6.4%) | 0.49 (0.44-0.54) | <0.001 | <0.001 |
|  | 95 plus | 274 (1.2%) | 46 (0.4%) | 0.3 (0.21-0.4) | <0.001 | <0.001 |
| Social status | 1 | 1004 (4.4%) | 457 (3.8%) |  | (reference level) | (reference level) |
|  | 2 | 15898 (70%) | 7861 (65.9%) | 1.09 (0.97-1.22) | 0.154 | 0.234 |
|  | 3 | 5643 (24.8%) | 3548 (29.7%) | 1.38 (1.23-1.56) | <0.001 | <0.001 |
|  | 4 | 177 (0.8%) | 68 (0.6%) | 0.84 (0.62-1.13) | 0.269 | 0.280 |
| Diabetes | 0 | 16907 (74.4%) | 8525 (71.4%) |  |  |  |
|  | 1 | 5815 (25.6%) | 3409 (28.6%) | 1.16 (1.11-1.22) | <0.001 | <0.001 |
| Neoplasm | 0 | 17747 (78.1%) | 8850 (74.2%) |  |  |  |
|  | 1 | 4975 (21.9%) | 3084 (25.8%) | 1.24 (1.18-1.31) | <0.001 | <0.001 |
| Asthma | 0 | 21284 (93.7%) | 11016 (92.3%) |  |  |  |
|  | 1 | 1438 (6.3%) | 918 (7.7%) | 1.23 (1.13-1.34) | <0.001 | <0.001 |
| Dementia | 0 | 17369 (76.4%) | 10228 (85.7%) |  |  |  |
|  | 1 | 5353 (23.6%) | 1706 (14.3%) | 0.54 (0.51-0.57) | <0.001 | <0.001 |
| Ictus | 0 | 18824 (82.8%) | 10058 (84.3%) |  |  |  |
|  | 1 | 3898 (17.2%) | 1876 (15.7%) | 0.9 (0.85-0.96) | 0.001 | 0.002 |
| Heart Failure | 0 | 19497 (85.8%) | 10023 (84%) |  |  |  |
|  | 1 | 3225 (14.2%) | 1911 (16%) | 1.15 (1.08-1.23) | <0.001 | <0.001 |
| Renal Insufficiency | 0 | 18958 (83.4%) | 9831 (82.4%) |  |  |  |
|  | 1 | 3764 (16.6%) | 2103 (17.6%) | 1.08 (1.02-1.14) | 0.013 | 0.017 |
| Ischemia | 0 | 20172 (88.8%) | 10282 (86.2%) |  |  |  |
|  | 1 | 2550 (11.2%) | 1652 (13.8%) | 1.27 (1.19-1.36) | <0.001 | <0.001 |
| COPD | 0 | 19995 (88%) | 10026 (84%) |  |  |  |
|  | 1 | 2727 (12%) | 1908 (16%) | 1.4 (1.31-1.49) | <0.001 | <0.001 |
| Hematological Neoplasm | 0 | 22514 (99.1%) | 11765 (98.6%) |  |  |  |
|  | 1 | 208 (0.9%) | 169 (1.4%) | 1.55 (1.27-1.91) | <0.001 | <0.001 |
| Obesity | 0 | 17324 (76.2%) | 8487 (71.1%) |  |  |  |
|  | 1 | 5398 (23.8%) | 3447 (28.9%) | 1.3 (1.24-1.37) | <0.001 | <0.001 |
| Smoking | 0 | 21227 (93.4%) | 10942 (91.7%) |  |  |  |
|  | 1 | 1495 (6.6%) | 992 (8.3%) | 1.29 (1.18-1.4) | <0.001 | <0.001 |
| Transplants | 0 | 22660 (99.7%) | 11841 (99.2%) |  |  |  |
|  | 1 | 62 (0.3%) | 93 (0.8%) | 2.87 (2.09-3.98) | <0.001 | <0.001 |
| Metabolic Syndrome | 0 | 14916 (65.6%) | 7580 (63.5%) |  |  |  |
|  | 1 | 7806 (34.4%) | 4354 (36.5%) | 1.1 (1.05-1.15) | <0.001 | <0.001 |
| Other Immunosuppressants | 0 | 22569 (99.3%) | 11808 (98.9%) |  |  |  |
|  | 1 | 153 (0.7%) | 126 (1.1%) | 1.57 (1.24-1.99) | <0.001 | <0.001 |
| Inhib Calcineurin | 0 | 22668 (99.8%) | 11849 (99.3%) |  |  |  |
|  | 1 | 54 (0.2%) | 85 (0.7%) | 3.01 (2.15-4.26) | <0.001 | <0.001 |
| Rituximab | 0 | 22709 (99.9%) | 11913 (99.8%) |  |  |  |
|  | 1 | 13 (0.1%) | 21 (0.2%) | 3.08 (1.56-6.31) | 0.001 | 0.001 |
| JAK Inhib | 0 | 22713 (100%) | 11920 (99.9%) |  |  |  |
|  | 1 | 9 (0%) | 14 (0.1%) | 2.96 (1.3-7.12) | 0.011 | 0.014 |
| Selective Immunosuppressants | 0 | 22629 (99.6%) | 11812 (99%) |  |  |  |
|  | 1 | 93 (0.4%) | 122 (1%) | 2.51 (1.92-3.3) | <0.001 | <0.001 |
| Systemic Corticosteroids | 0 | 21943 (96.6%) | 11247 (94.2%) |  |  |  |
|  | 1 | 779 (3.4%) | 687 (5.8%) | 1.72 (1.55-1.91) | <0.001 | <0.001 |
| Metformin | 0 | 20384 (89.7%) | 10573 (88.6%) |  |  |  |
|  | 1 | 2338 (10.3%) | 1361 (11.4%) | 1.12 (1.05-1.2) | 0.001 | 0.002 |
| Inhib DPP4 | 0 | 22096 (97.2%) | 11490 (96.3%) |  |  |  |
|  | 1 | 626 (2.8%) | 444 (3.7%) | 1.36 (1.2-1.54) | <0.001 | <0.001 |
| VitK Antagonists | 0 | 21608 (95.1%) | 11236 (94.2%) |  |  |  |
|  | 1 | 1114 (4.9%) | 698 (5.8%) | 1.2 (1.09-1.33) | <0.001 | <0.001 |
| Heparins | 0 | 22431 (98.7%) | 11727 (98.3%) |  |  |  |
|  | 1 | 291 (1.3%) | 207 (1.7%) | 1.36 (1.14-1.63) | 0.001 | 0.001 |
| Alpha1 adrenergic inhib | 0 | 20584 (90.6%) | 10207 (85.5%) |  |  |  |
|  | 1 | 2138 (9.4%) | 1727 (14.5%) | 1.63 (1.52-1.74) | <0.001 | <0.001 |
| ACEi combinations | 0 | 17596 (77.4%) | 9063 (75.9%) |  |  |  |
|  | 1 | 5126 (22.6%) | 2871 (24.1%) | 1.09 (1.03-1.15) | 0.002 | 0.003 |
| ARB combinations | 0 | 19143 (84.2%) | 9775 (81.9%) |  |  |  |
|  | 1 | 3579 (15.8%) | 2159 (18.1%) | 1.18 (1.11-1.25) | <0.001 | <0.001 |
| HMCoA reductase | 0 | 16694 (73.5%) | 7905 (66.2%) |  |  |  |
|  | 1 | 6028 (26.5%) | 4029 (33.8%) | 1.41 (1.35-1.48) | <0.001 | <0.001 |
| Cox2 Inhib Antiaggregants | 0 | 22722 (100%) | 11931 (100%) |  |  |  |
|  | 1 | 0 (0%) | 3 (0%) | 73881.76 (1.42-) | 0.871 | 0.786 |
| Num drugs patient |  | 1.2 (1.3) | 1.4 (1.3) | 1.16 (1.14-1.18) | <0.001 | <0.001 |
| Polypharmacy |  | 0.0031 (0.055) | 0.0049 (0.07) | 1.61 (1.13-2.27) | 0.007 | 0.010 |
| GMA: Adjusted morbidity groups. Social status: 1) exempted (non-working population or non-contributory pension recipients); 2) < 18,000€ income per year; 3) 18,000€ to 100,000€ income per year; and 4) > 100,000€ per year. ACEi: Angiotensin-converting-enzyme inhibitors, ARB: Angiotensin receptor blockers, DPP4: Dipeptidyl peptidase 4, HIV: human immunodeficiency virus, HMCoA: ß-Hydroxy ß-methylglutaryl-CoA, Inhib: inhibitors, COPD: Chronic obstructive pulmonary disease, TI: reverse transcriptase, TNF: Tumor necrosis factor, Vit K: vitamine K. The adjusted p-value was computed using the Benjamini-Yekutieli (BY) correction method. | | | | | | |

**Supplementary Material 4. Socio-demographic and health characteristics of deceased participants aged 18 to 65 years in the cohort studied.** Odds Ratio (OR) estimates and 95% confidence intervals (CI) for significant socio-demographic, chronic illness, and medication variables in univariate logistic regression for the risk of mortality in participants diagnosed with COVID-19. Data are presented as the total number of participants and the percentage (%) in relation to the overall cohort for categorical variables, and mean and standard deviation (SD) for numerical variables, using a univariate model by age groups (18 to 65 years old). Probabilities were calculated using the Student’s t-test for continuous variables and chi-squared for categorical variables (N = 85,994). The reference group for disease and drug variables was “No”.

| **Variable** | **Levels** | **Not deceased 85449 (99.37%)** | **Deceased 545 (0.63%)** | **OR (CI)** | **Pr(>\|z\|)** | **Adjusted-p-value BY** |
| --- | --- | --- | --- | --- | --- | --- |
| Sex | Female | 47830 (56%) | 171 (31.4%) | 1 | (reference level) | (reference level) |
|  | Male | 37619 (44%) | 374 (68.6%) | 2.78 (2.32-3.34) | <0.001 | <0.001 |
| Age at exposure | 20-24 | 7453 (8.7%) | 3 (0.6%) | 1 | (reference level) | (reference level) |
|  | 25-29 | 8414 (9.8%) | 4 (0.7%) | 1.18 (0.26-6) | 0.828 | 0.54 |
|  | 30-34 | 8059 (9.4%) | 4 (0.7%) | 1.23 (0.27-6.26) | 0.784 | 0.463 |
|  | 35-39 | 9696 (11.3%) | 9 (1.7%) | 2.31 (0.69-10.4) | 0.21 | 0.09 |
|  | 40-44 | 11449 (13.4%) | 22 (4%) | 4.77 (1.65-20.17) | 0.011 | 0.015 |
|  | 45-49 | 11479 (13.4%) | 29 (5.3%) | 6.28 (2.23-26.22) | 0.002 | 0.003 |
|  | 50-54 | 10922 (12.8%) | 85 (15.6%) | 19.33 (7.25-78.79) | <0.001 | <0.001 |
|  | 55-59 | 9943 (11.6%) | 149 (27.3%) | 37.23 (14.13-150.88) | <0.001 | <0.001 |
|  | 60-64 | 8034 (9.4%) | 240 (44%) | 74.21 (28.33-299.93) | <0.001 | <0.001 |
| Social status | 1 | 2960 (3.5%) | 65 (11.9%) | 1 | (reference level) | (reference level) |
|  | 2 | 51567 (60.3%) | 325 (59.6%) | 0.29 (0.22-0.38) | <0.001 | <0.001 |
|  | 3 | 30059 (35.2%) | 153 (28.1%) | 0.23 (0.17-0.31) | <0.001 | <0.001 |
|  | 4 | 863 (1%) | 2 (0.4%) | 0.11 (0.02-0.34) | 0.002 | 0.004 |
| GMA Cat | 1. Very low risk | 34892 (40.8%) | 105 (19.3%) | 1 | (reference level) | (reference level) |
|  | 2. Low risk | 22479 (26.3%) | 124 (22.8%) | 1.83 (1.41-2.38) | <0.001 | <0.001 |
|  | 3. Moderate risk | 12831 (15%) | 114 (20.9%) | 2.95 (2.26-3.86) | <0.001 | <0.001 |
|  | 4. High risk | 9975 (11.7%) | 103 (18.9%) | 3.43 (2.61-4.51) | <0.001 | <0.001 |
|  | 5. Very high risk | 5272 (6.2%) | 99 (18.2%) | 6.24 (4.73-8.22) | <0.001 | <0.001 |
| Diabetes | 0 | 81093 (94.9%) | 425 (78%) | 1 | (reference level) | (reference level) |
|  | 1 | 4356 (5.1%) | 120 (22%) | 5.26 (4.27-6.43) | <0.001 | <0.001 |
| Hypertension | 0 | 76952 (90.1%) | 385 (70.6%) | 1 | (reference level) | (reference level) |
|  | 1 | 8497 (9.9%) | 160 (29.4%) | 3.76 (3.12-4.52) | <0.001 | <0.001 |
| Neoplasm | 0 | 82285 (96.3%) | 421 (77.2%) | 1 | (reference level) | (reference level) |
|  | 1 | 3164 (3.7%) | 124 (22.8%) | 7.66 (6.23-9.36) | <0.001 | <0.001 |
| Dementia | 0 | 85175 (99.7%) | 522 (95.8%) | 1 | (reference level) | (reference level) |
|  | 1 | 274 (0.3%) | 23 (4.2%) | 13.7 (8.64-20.67) | <0.001 | <0.001 |
| Ictus | 0 | 84141 (98.5%) | 502 (92.1%) | 1 | (reference level) | (reference level) |
|  | 1 | 1308 (1.5%) | 43 (7.9%) | 5.51 (3.96-7.47) | <0.001 | <0.001 |
| Heart Failure | 0 | 85018 (99.5%) | 504 (92.5%) | 1 | (reference level) | (reference level) |
|  | 1 | 431 (0.5%) | 41 (7.5%) | 16.05 (11.35-22.09) | <0.001 | <0.001 |
| Renal Insufficiency | 0 | 84715 (99.1%) | 500 (91.7%) | 1 | (reference level) | (reference level) |
|  | 1 | 734 (0.9%) | 45 (8.3%) | 10.39 (7.49-14.05) | <0.001 | <0.001 |
| Ischemia | 0 | 84292 (98.6%) | 486 (89.2%) | 1 | (reference level) | (reference level) |
|  | 1 | 1157 (1.4%) | 59 (10.8%) | 8.84 (6.64-11.56) | <0.001 | <0.001 |
| COPD | 0 | 84212 (98.6%) | 472 (86.6%) | 1 | (reference level) | (reference level) |
|  | 1 | 1237 (1.4%) | 73 (13.4%) | 10.53 (8.12-13.47) | <0.001 | <0.001 |
| Hematological Neoplasm | 0 | 85225 (99.7%) | 537 (98.5%) | 1 | (reference level) | (reference level) |
|  | 1 | 224 (0.3%) | 8 (1.5%) | 5.67 (2.56-10.78) | <0.001 | <0.001 |
| Obesity | 0 | 73579 (86.1%) | 371 (68.1%) | 1 | (reference level) | (reference level) |
|  | 1 | 11870 (13.9%) | 174 (31.9%) | 2.91 (2.42-3.48) | <0.001 | <0.001 |
| Smoking | 0 | 78095 (91.4%) | 437 (80.2%) | 1 | (reference level) | (reference level) |
|  | 1 | 7354 (8.6%) | 108 (19.8%) | 2.62 (2.11-3.23) | <0.001 | <0.001 |
| Transplants | 0 | 85282 (99.8%) | 530 (97.2%) | 1 | (reference level) | (reference level) |
|  | 1 | 167 (0.2%) | 15 (2.8%) | 14.45 (8.12-23.85) | <0.001 | <0.001 |
| Metabolic Syndrome | 0 | 76430 (89.4%) | 405 (74.3%) | 1 | (reference level) | (reference level) |
|  | 1 | 9019 (10.6%) | 140 (25.7%) | 2.93 (2.41-3.54) | <0.001 | <0.001 |
| HIV | 0 | 84778 (99.2%) | 535 (98.2%) | 1 | (reference level) | (reference level) |
|  | 1 | 671 (0.8%) | 10 (1.8%) | 2.36 (1.17-4.19) | 0.008 | 0.010 |
| Inhib Calcineurin | 0 | 85253 (99.8%) | 525 (96.3%) | 1 | (reference level) | (reference level) |
|  | 1 | 196 (0.2%) | 20 (3.7%) | 16.57 (10.07-25.8) | <0.001 | <0.001 |
| Rituximab | 0 | 85424 (100%) | 542 (99.4%) | 1 | (reference level) | (reference level) |
|  | 1 | 25 (0%) | 3 (0.6%) | 18.91 (4.49-54.1) | <0.001 | <0.001 |
| Selective Immunosuppressants | 0 | 85184 (99.7%) | 522 (95.8%) | 1 | (reference level) | (reference level) |
|  | 1 | 265 (0.3%) | 23 (4.2%) | 14.16 (8.93-21.39) | <0.001 | <0.001 |
| Systemic Corticosteroids | 0 | 84691 (99.1%) | 479 (87.9%) | 1 | (reference level) | (reference level) |
|  | 1 | 758 (0.9%) | 66 (12.1%) | 15.39 (11.69-19.96) | <0.001 | <0.001 |
| Metformin | 0 | 83916 (98.2%) | 504 (92.5%) | 1 | (reference level) | (reference level) |
|  | 1 | 1533 (1.8%) | 41 (7.5%) | 4.45 (3.18-6.07) | <0.001 | <0.001 |
| Inhib DPP4 | 0 | 85216 (99.7%) | 536 (98.3%) | 1 | (reference level) | (reference level) |
|  | 1 | 233 (0.3%) | 9 (1.7%) | 6.14 (2.91-11.31) | <0.001 | <0.001 |
| Direct Action Oral Anticoagulants | 0 | 85304 (99.8%) | 536 (98.3%) | 1 | (reference level) | (reference level) |
|  | 1 | 145 (0.2%) | 9 (1.7%) | 9.88 (4.65-18.37) | <0.001 | <0.001 |
| VitK Antagonists | 0 | 85221 (99.7%) | 533 (97.8%) | 1 | (reference level) | (reference level) |
|  | 1 | 228 (0.3%) | 12 (2.2%) | 8.42 (4.43-14.47) | <0.001 | <0.001 |
| Heparins | 0 | 84978 (99.4%) | 533 (97.8%) | 1 | (reference level) | (reference level) |
|  | 1 | 471 (0.6%) | 12 (2.2%) | 4.06 (2.15-6.92) | <0.001 | <0.001 |
| Alpha1 adrenergic inhib | 0 | 84752 (99.2%) | 503 (92.3%) | 1 | (reference level) | (reference level) |
|  | 1 | 697 (0.8%) | 42 (7.7%) | 10.15 (7.24-13.86) | <0.001 | <0.001 |
| ACEi combinations | 0 | 81668 (95.6%) | 476 (87.3%) | 1 | (reference level) | (reference level) |
|  | 1 | 3781 (4.4%) | 69 (12.7%) | 3.13 (2.41-4.01) | <0.001 | <0.001 |
| ARB combinations | 0 | 83323 (97.5%) | 499 (91.6%) | 1 | (reference level) | (reference level) |
|  | 1 | 2126 (2.5%) | 46 (8.4%) | 3.61 (2.63-4.84) | <0.001 | <0.001 |
| HMCoA reductase | 0 | 81934 (95.9%) | 437 (80.2%) | 1 | (reference level) | (reference level) |
|  | 1 | 3515 (4.1%) | 108 (19.8%) | 5.76 (4.63-7.1) | <0.001 | <0.001 |
| Inhib TI nucleosides and nucleotides | 0 | 85272 (99.8%) | 541 (99.3%) | 1 | (reference level) | (reference level) |
|  | 1 | 177 (0.2%) | 4 (0.7%) | 3.56 (1.09-8.44) | 0.012 | 0.034 |
| Inhib Integrase | 0 | 85130 (99.6%) | 539 (98.9%) | 1 | (reference level) | (reference level) |
|  | 1 | 319 (0.4%) | 6 (1.1%) | 2.97 (1.17-6.11) | 0.009 | 0.012 |
| Num drugs patient |  | 0.2 (0.62) | 0.96 (1.3) | 2 (1.89-2.12) | <0.001 | <0.001 |
| Polypharmacy |  | 0.0034 (0.018) | 0.0055 (0.074) | 16.3 (3.9-46.04) | <0.001 | <0.001 |
| GMA: Adjusted morbidity groups. Social status: 1) exempted (non-working population or non-contributory pension recipients); 2) < 18,000€ income per year; 3) 18,000€ to 100,000€ income per year; and 4) > 100,000€ per year. ACEi: Angiotensin-converting-enzyme inhibitors, ARB: Angiotensin receptor blockers, DPP4: Dipeptidyl peptidase 4, HIV: human immunodeficiency virus, HMCoA: ß-Hydroxy ß-methylglutaryl-CoA, Inhib: inhibitors, COPD: Chronic obstructive pulmonary disease, TI: reverse transcriptase, TNF: Tumor necrosis factor, Vit K: vitamine K. The adjusted p-value was computed using the Benjamini-Yekutieli (BY) correction method. | | | | | |  |

**Supplementary Material 5. Socio-demographic and health characteristics of deceased participants over 65 years in the cohort studied.** Odds Ratio (OR) estimates and 95% confidence intervals (CI) for significant socio-demographic, chronic illness, and medication variables in univariate logistic regression for the risk of mortality in participants diagnosed with COVID-19. Data are presented as the total number of participants and the percentage (%) in relation to the overall cohort for categorical variables, and mean and standard deviation (SD) for numerical variables, using a univariate model by age groups (18 to 65 years old). Probabilities were calculated using the Student’s t-test for continuous variables and chi-squared for categorical variables (N = 34,656). The reference group for disease and drug variables was “No”.

| **Variable** | **Levels** | **Not deceased 28832 (83.19%)** | **Deceased 5824 (16.81%)** | **OR (CI)** | **Pr(>\|z\|)** | **Adjusted-p-val BY** |
| --- | --- | --- | --- | --- | --- | --- |
| Sex | Female | 17568 (60.9%) | 2959 (50.8%) | 1 | (reference level) | (reference level) |
|  | Male | 11264 (39.1%) | 2865 (49.2%) | 1.51 (1.43-1.6) | <0.001 | <0.001 |
| Age at exposure | 65-69 | 5540 (19.2%) | 402 (6.9%) | 1 | (reference level) | (reference level) |
|  | 70-74 | 5268 (18.3%) | 645 (11.1%) | 1.69 (1.48-1.92) | <0.001 | <0.001 |
|  | 75-79 | 5011 (17.4%) | 993 (17.1%) | 2.73 (2.42-3.09) | <0.001 | <0.001 |
|  | 80-84 | 4689 (16.3%) | 1322 (22.7%) | 3.89 (3.46-4.38) | <0.001 | <0.001 |
|  | 85-89 | 5345 (18.5%) | 1608 (27.6%) | 4.15 (3.7-4.66) | <0.001 | <0.001 |
|  | 90-94 | 2728 (9.5%) | 785 (13.5%) | 3.97 (3.49-4.51) | <0.001 | <0.001 |
|  | 95 plus | 251 (0.9%) | 69 (1.2%) | 3.79 (2.83-5.01) | <0.001 | <0.001 |
| Social status | 1 | 1280 (4.4%) | 181 (3.1%) | 1 | (reference level) | (reference level) |
|  | 2 | 19574 (67.9%) | 4185 (71.9%) | 1.51 (1.29-1.78) | <0.001 | <0.001 |
|  | 3 | 7758 (26.9%) | 1433 (24.6%) | 1.31 (1.11-1.55) | 0.002 | 0.004 |
|  | 4 | 220 (0.8%) | 25 (0.4%) | 0.8 (0.51-1.23) | 0.332 | 0.343 |
| GMA Cat | 1. Very low risk | 3275 (11.4%) | 353 (6.1%) | 1 | (reference level) | (reference level) |
|  | 2. Low risk | 7519 (26.1%) | 1121 (19.2%) | 1.38 (1.22-1.57) | <0.001 | <0.001 |
|  | 3. Moderate risk | 6350 (22%) | 1211 (20.8%) | 1.77 (1.56-2.01) | <0.001 | <0.001 |
|  | 4. High risk | 6939 (24.1%) | 1670 (28.7%) | 2.23 (1.98-2.53) | <0.001 | <0.001 |
|  | 5. Very high risk | 4749 (16.5%) | 1469 (25.2%) | 2.87 (2.54-3.25) | <0.001 | <0.001 |
| Diabetes | 0 | 21489 (74.5%) | 3943 (67.7%) | 1 | (reference level) | (reference level) |
|  | 1 | 7343 (25.5%) | 1881 (32.3%) | 1.4 (1.31-1.48) | <0.001 | <0.001 |
| Hypertension | 0 | 13972 (48.5%) | 2502 (43%) | 1 | (reference level) | (reference level) |
|  | 1 | 14860 (51.5%) | 3322 (57%) | 1.25 (1.18-1.32) | <0.001 | <0.001 |
| Neoplasm | 0 | 22362 (77.6%) | 4235 (72.7%) | 1 | (reference level) | (reference level) |
|  | 1 | 6470 (22.4%) | 1589 (27.3%) | 1.3 (1.22-1.38) | <0.001 | <0.001 |
| Dementia | 0 | 23334 (80.9%) | 4263 (73.2%) | 1 | (reference level) | (reference level) |
|  | 1 | 5498 (19.1%) | 1561 (26.8%) | 1.55 (1.46-1.66) | <0.001 | <0.001 |
| Ictus | 0 | 24296 (84.3%) | 4586 (78.7%) | 1 | (reference level) | (reference level) |
|  | 1 | 4536 (15.7%) | 1238 (21.3%) | 1.45 (1.35-1.55) | <0.001 | <0.001 |
| Heart Failure | 0 | 24973 (86.6%) | 4547 (78.1%) | 1 | (reference level) | (reference level) |
|  | 1 | 3859 (13.4%) | 1277 (21.9%) | 1.82 (1.69-1.95) | <0.001 | <0.001 |
| Renal Insufficiency | 0 | 24366 (84.5%) | 4423 (75.9%) | 1 | (reference level) | (reference level) |
|  | 1 | 4466 (15.5%) | 1401 (24.1%) | 1.73 (1.61-1.85) | <0.001 | <0.001 |
| Ischemia | 0 | 25520 (88.5%) | 4934 (84.7%) | 1 | (reference level) | (reference level) |
|  | 1 | 3312 (11.5%) | 890 (15.3%) | 1.39 (1.28-1.51) | <0.001 | <0.001 |
| COPD | 0 | 25260 (87.6%) | 4761 (81.7%) | 1 | (reference level) | (reference level) |
|  | 1 | 3572 (12.4%) | 1063 (18.3%) | 1.58 (1.46-1.7) | <0.001 | <0.001 |
| Hematological Neoplasm | 0 | 28543 (99%) | 5736 (98.5%) | 1 | (reference level) | (reference level) |
|  | 1 | 289 (1%) | 88 (1.5%) | 1.52 (1.19-1.92) | 0.001 | 0.69 |
| Transplants | 0 | 28720 (99.6%) | 5781 (99.3%) | 1 | (reference level) | (reference level) |
|  | 1 | 112 (0.4%) | 43 (0.7%) | 1.91 (1.33-2.69) | <0.001 | <0.001 |
| Metabolic Syndrome | 0 | 18611 (64.5%) | 3885 (66.7%) | 1 | (reference level) | (reference level) |
|  | 1 | 10221 (35.5%) | 1939 (33.3%) | 0.91 (0.86-0.96) | 0.002 | 0.002 |
| Inhib Calcineurin | 0 | 28735 (99.7%) | 5782 (99.3%) | 1 | (reference level) | (reference level) |
|  | 1 | 97 (0.3%) | 42 (0.7%) | 2.15 (1.48-3.07) | <0.001 | <0.001 |
| Selective Immunosuppressants | 0 | 28678 (99.5%) | 5763 (99%) | 1 | (reference level) | (reference level) |
|  | 1 | 154 (0.5%) | 61 (1%) | 1.97 (1.45-2.64) | <0.001 | <0.001 |
| Systemic Corticosteroids | 0 | 27744 (96.2%) | 5446 (93.5%) | 1 | (reference level) | (reference level) |
|  | 1 | 1088 (3.8%) | 378 (6.5%) | 1.77 (1.57-1.99) | <0.001 | <0.001 |
| Inhib DPP4 | 0 | 28007 (97.1%) | 5579 (95.8%) | 1 | (reference level) | (reference level) |
|  | 1 | 825 (2.9%) | 245 (4.2%) | 1.49 (1.29-1.72) | <0.001 | <0.001 |
| Direct Action Oral Anticoagulants | 0 | 27290 (94.7%) | 5421 (93.1%) | 1 | (reference level) | (reference level) |
|  | 1 | 1542 (5.3%) | 403 (6.9%) | 1.32 (1.17-1.47) | <0.001 | <0.001 |
| VitK Antagonists | 0 | 27385 (95%) | 5459 (93.7%) | 1 | (reference level) | (reference level) |
|  | 1 | 1447 (5%) | 365 (6.3%) | 1.27 (1.12-1.42) | <0.001 | <0.001 |
| Alpha1 adrenergic inhib | 0 | 25785 (89.4%) | 5006 (86%) | 1 | (reference level) | (reference level) |
|  | 1 | 3047 (10.6%) | 818 (14%) | 1.38 (1.27-1.5) | <0.001 | <0.001 |
| Num drugs patient |  | 1.2 (1.3) | 1.3 (1.3) | 1.08 (1.05-1.1) | <0.001 | <0.001 |
| GMA: Adjusted morbidity groups. Social status: 1) exempted (non-working population or non-contributory pension recipients); 2) < 18,000€ income per year; 3) 18,000€ to 100,000€ income per year; and 4) > 100,000€ per year. ACEi: Angiotensin-converting-enzyme inhibitors, ARB: Angiotensin receptor blockers, DPP4: Dipeptidyl peptidase 4, HIV: human immunodeficiency virus, HMCoA: ß-Hydroxy ß-methylglutaryl-CoA, Inhib: inhibitors, COPD: Chronic obstructive pulmonary disease, TI: reverse transcriptase, TNF: Tumor necrosis factor, Vit K: vitamine K. The adjusted p-value was computed using the Benjamini-Yekutieli (BY) correction method. | | | | | |  |

**Supplementary Material 6. Socio-demographic and health characteristics of participants admitted in ICU aged 18 to 65 years in the cohort studied.** Odds Ratio (OR) estimates and 95% confidence intervals (CI) for significant socio-demographic, chronic illness, and medication variables in univariate logistic regression for the risk of ICU admission in participants diagnosed with COVID-19. Data are presented as the total number of participants and the percentage (%) in relation to the overall cohort for categorical variables, and mean and standard deviation (SD) for numerical variables, using a univariate model by age groups (18 to 65 years old). Probabilities were calculated using the Student’s t-test for continuous variables and chi-squared for categorical variables (N = 85,994). The reference group for disease and drug variables was “No”.

| **Variable** | **Levels** | **No ICU admission 84750 (98.55%)** | **ICU admission 1244 (1.45%)** | **OR (CI)** | **Pr(>\|z\|)** | **Adjusted-p-val BY** |
| --- | --- | --- | --- | --- | --- | --- |
| Sex | Female | 47634 (56.2%) | 367 (29.5%) | 1 | (reference level) | (reference level) |
|  | Male | 37116 (43.8%) | 877 (70.5%) | 3.07 (2.72-3.47) | <0.001 | <0.001 |
| Age at exposure | 20-24 | 7438 (8.8%) | 18 (1.4%) | 1 | (reference level) | (reference level) |
|  | 25-29 | 8393 (9.9%) | 25 (2%) | 1.23 (0.67-2.29) | 0.502 | 0.4152 |
|  | 30-34 | 8024 (9.5%) | 39 (3.1%) | 2.01 (1.17-3.6) | 0.015 | 0.014 |
|  | 35-39 | 9650 (11.4%) | 55 (4.4%) | 2.36 (1.41-4.13) | 0.002 | 0.002 |
|  | 40-44 | 11377 (13.4%) | 94 (7.6%) | 3.41 (2.11-5.84) | <0.001 | <0.001 |
|  | 45-49 | 11357 (13.4%) | 151 (12.1%) | 5.49 (3.46-9.28) | <0.001 | <0.001 |
|  | 50-54 | 10784 (12.7%) | 223 (17.9%) | 8.54 (5.44-14.33) | <0.001 | <0.001 |
|  | 55-59 | 9806 (11.6%) | 286 (23%) | 12.05 (7.71-20.14) | <0.001 | <0.001 |
|  | 60-64 | 7921 (9.3%) | 353 (28.4%) | 18.42 (11.82-30.71) | <0.001 | <0.001 |
| Social status | 1 | 2942 (3.5%) | 83 (6.7%) | 1 | (reference level) | (reference level) |
|  | 2 | 51196 (60.4%) | 696 (55.9%) | 0.48 (0.39-0.61) | <0.001 | <0.001 |
|  | 3 | 29765 (35.1%) | 447 (35.9%) | 0.53 (0.42-0.68) | <0.001 | <0.001 |
|  | 4 | 847 (1%) | 18 (1.4%) | 0.75 (0.44-1.23) | 0.281 | 0.317 |
| GMA Cat | 1. Very low risk | 34611 (40.8%) | 386 (31%) | 1 | (reference level) | (reference level) |
|  | 2. Low risk | 22258 (26.3%) | 345 (27.7%) | 1.39 (1.2-1.61) | <0.001 | <0.001 |
|  | 3. Moderate risk | 12717 (15%) | 228 (18.3%) | 1.61 (1.36-1.89) | <0.001 | <0.001 |
|  | 4. High risk | 9891 (11.7%) | 187 (15%) | 1.7 (1.42-2.02) | <0.001 | <0.001 |
|  | 5. Very high risk | 5273 (6.2%) | 98 (7.9%) | 1.67 (1.33-2.07) | <0.001 | <0.001 |
| Diabetes | 0 | 80494 (95%) | 1024 (82.3%) | 1 | (reference level) | (reference level) |
|  | 1 | 4256 (5%) | 220 (17.7%) | 4.06 (3.49-4.71) | <0.001 | <0.001 |
| Hypertension | 0 | 76420 (90.2%) | 917 (73.7%) | 1 | (reference level) | (reference level) |
|  | 1 | 8330 (9.8%) | 327 (26.3%) | 3.27 (2.87-3.71) | <0.001 | <0.001 |
| Neoplasm | 0 | 81554 (96.2%) | 1152 (92.6%) | 1 | (reference level) | (reference level) |
|  | 1 | 3196 (3.8%) | 92 (7.4%) | 2.04 (1.63-2.51) | <0.001 | <0.001 |
| Ictus | 0 | 83476 (98.5%) | 1167 (93.8%) | 1 | (reference level) | (reference level) |
|  | 1 | 1274 (1.5%) | 77 (6.2%) | 4.32 (3.38-5.44) | <0.001 | <0.001 |
| Heart Failure | 0 | 84316 (99.5%) | 1206 (96.9%) | 1 | (reference level) | (reference level) |
|  | 1 | 434 (0.5%) | 38 (3.1%) | 6.12 (4.3-8.45) | <0.001 | <0.001 |
| Renal Insufficiency | 0 | 84026 (99.1%) | 1189 (95.6%) | 1 | (reference level) | (reference level) |
|  | 1 | 724 (0.9%) | 55 (4.4%) | 5.37 (4.01-7.03) | <0.001 | <0.001 |
| Ischemia | 0 | 83622 (98.7%) | 1156 (92.9%) | 1 | (reference level) | (reference level) |
|  | 1 | 1128 (1.3%) | 88 (7.1%) | 5.64 (4.48-7.02) | <0.001 | <0.001 |
| COPD | 0 | 83491 (98.5%) | 1193 (95.9%) | 1 | (reference level) | (reference level) |
|  | 1 | 1259 (1.5%) | 51 (4.1%) | 2.83 (2.1-3.73) | <0.001 | <0.001 |
| Obesity | 0 | 73080 (86.2%) | 870 (69.9%) | 1 | (reference level) | (reference level) |
|  | 1 | 11670 (13.8%) | 374 (30.1%) | 2.69 (2.38-3.04) | <0.001 | <0.001 |
| Smoking | 0 | 77431 (91.4%) | 1101 (88.5%) | 1 | (reference level) | (reference level) |
|  | 1 | 7319 (8.6%) | 143 (11.5%) | 1.37 (1.15-1.63) | <0.001 | <0.001 |
| Transplants | 0 | 84584 (99.8%) | 1228 (98.7%) | 1 | (reference level) | (reference level) |
|  | 1 | 166 (0.2%) | 16 (1.3%) | 6.64 (3.81-10.77) | <0.001 | <0.001 |
| Metabolic Syndrome | 0 | 75890 (89.5%) | 945 (76%) | 1 | (reference level) | (reference level) |
|  | 1 | 8860 (10.5%) | 299 (24%) | 2.71 (2.37-3.09) | <0.001 | <0.001 |
| Inhib Calcineurin | 0 | 84558 (99.8%) | 1220 (98.1%) | 1 | (reference level) | (reference level) |
|  | 1 | 192 (0.2%) | 24 (1.9%) | 8.66 (5.51-13.02) | <0.001 | <0.001 |
| Rituximab | 0 | 84727 (100%) | 1239 (99.6%) | 1 | (reference level) | (reference level) |
|  | 1 | 23 (0%) | 5 (0.4%) | 14.87 (4.99-36.13) | <0.001 | <0.001 |
| Selective Immunosuppressants | 0 | 84487 (99.7%) | 1219 (98%) | 1 | (reference level) | (reference level) |
|  | 1 | 263 (0.3%) | 25 (2%) | 6.59 (4.25-9.76) | <0.001 | <0.001 |
| Systemic Corticosteroids | 0 | 83967 (99.1%) | 1203 (96.7%) | 1 | (reference level) | (reference level) |
|  | 1 | 783 (0.9%) | 41 (3.3%) | 3.65 (2.62-4.96) | <0.001 | <0.001 |
| Metformin | 0 | 83243 (98.2%) | 1177 (94.6%) | 1 | (reference level) | (reference level) |
|  | 1 | 1507 (1.8%) | 67 (5.4%) | 3.14 (2.42-4.01) | <0.001 | <0.001 |
| Inhib DPP4 | 0 | 84518 (99.7%) | 1234 (99.2%) | 1 | (reference level) | (reference level) |
|  | 1 | 232 (0.3%) | 10 (0.8%) | 2.95 (1.46-5.28) | 0.001 | 0.001 |
| Direct Action Oral Anticoagulants | 0 | 84612 (99.8%) | 1228 (98.7%) | 1 | (reference level) | (reference level) |
|  | 1 | 138 (0.2%) | 16 (1.3%) | 7.99 (4.57-13.03) | <0.001 | <0.001 |
| VitK Antagonists | 0 | 84523 (99.7%) | 1231 (99%) | 1 | (reference level) | (reference level) |
|  | 1 | 227 (0.3%) | 13 (1%) | 3.93 (2.13-6.61) | <0.001 | <0.001 |
| Heparins | 0 | 84290 (99.5%) | 1221 (98.2%) | 1 | (reference level) | (reference level) |
|  | 1 | 460 (0.5%) | 23 (1.8%) | 3.45 (2.2-5.14) | <0.001 | <0.001 |
| Alpha1 adrenergic inhib | 0 | 84061 (99.2%) | 1194 (96%) | 1 | (reference level) | (reference level) |
|  | 1 | 689 (0.8%) | 50 (4%) | 5.11 (3.77-6.77) | <0.001 | <0.001 |
| ACEi combinations | 0 | 81039 (95.6%) | 1105 (88.8%) | 1 | (reference level) | (reference level) |
|  | 1 | 3711 (4.4%) | 139 (11.2%) | 2.75 (2.29-3.27) | <0.001 | <0.001 |
| ARB combinations | 0 | 82675 (97.6%) | 1147 (92.2%) | 1 | (reference level) | (reference level) |
|  | 1 | 2075 (2.4%) | 97 (7.8%) | 3.37 (2.71-4.14) | <0.001 | <0.001 |
| HMCoA reductase | 0 | 81300 (95.9%) | 1071 (86.1%) | 1 | (reference level) | (reference level) |
|  | 1 | 3450 (4.1%) | 173 (13.9%) | 3.81 (3.22-4.47) | <0.001 | <0.001 |
| Antimalarials | 0 | 84616 (99.8%) | 1236 (99.4%) | 1 | (reference level) | (reference level) |
|  | 1 | 134 (0.2%) | 8 (0.6%) | 4.09 (1.83-7.82) | <0.001 | <0.001 |
| Num drugs patient |  | 0.2 (0.61) | 0.63 (1.1) | 1.7 (1.62-1.78) | <0.001 | <0.001 |
| Polypharmacy |  | 0.00034 (0.018) | 0.0024 (0.049) | 7.06 (1.69-19.9) | 0.001 | 0.002 |
| GMA: Adjusted morbidity groups. Social status: 1) exempted (non-working population or non-contributory pension recipients); 2) < 18,000€ income per year; 3) 18,000€ to 100,000€ income per year; and 4) > 100,000€ per year. ACEi: Angiotensin-converting-enzyme inhibitors, ARB: Angiotensin receptor blockers, DPP4: Dipeptidyl peptidase 4, HIV: human immunodeficiency virus, HMCoA: ß-Hydroxy ß-methylglutaryl-CoA, Inhib: inhibitors, COPD: Chronic obstructive pulmonary disease, TI: reverse transcriptase, TNF: Tumor necrosis factor, Vit K: vitamine K. The adjusted p-value was computed using the Benjamini-Yekutieli (BY) correction method. | | | | | |  |

**Supplementary Material 7. Socio-demographic and health characteristics of participants admitted in ICU over 65 years in the cohort studied.** Odds Ratio (OR) estimates and 95% confidence intervals (CI) for significant socio-demographic, chronic illness, and medication variables in univariate logistic regression for the risk of ICU admission in participants diagnosed with COVID-19. Data are presented as the total number of participants and the percentage (%) in relation to the overall cohort for categorical variables, and mean and standard deviation (SD) for numerical variables, using a univariate model by age groups (18 to 65 years old). Probabilities were calculated using the Student’s t-test for continuous variables and chi-squared for categorical variables (N = 34,656). The reference group for disease and drug variables was “No”.

| **Variable** | **Levels** | **Not admitted ICU 33581 (96.9%)** | **Admitted ICU 1075 (3.1%)** | **OR (CI)** | **Pr(>\|z\|)** | **Adjusted-p-val BY** |
| --- | --- | --- | --- | --- | --- | --- |
| Sex | Female | 20169 (60.1%) | 358 (33.3%) | 1 | (reference level) | (reference level) |
|  | Male | 13412 (39.9%) | 717 (66.7%) | 3.01 (2.65-3.43) | <0.001 | <0.001 |
| Age at exposure | 65-69 | 5560 (16.6%) | 382 (35.5%) | 1 | (reference level) | (reference level) |
|  | 70-74 | 5568 (16.6%) | 345 (32.1%) | 0.9 (0.78-1.05) | 0.178 | 0.201 |
|  | 75-79 | 5775 (17.2%) | 229 (21.3%) | 0.58 (0.49-0.68) | <0.001 | <0.001 |
|  | 80-84 | 5938 (17.7%) | 73 (6.8%) | 0.18 (0.14-0.23) | <0.001 | <0.001 |
|  | 85-89 | 6912 (20.6%) | 41 (3.8%) | 0.09 (0.06-0.12) | <0.001 | <0.001 |
|  | 90-94 | 3508 (10.4%) | 5 (0.5%) | 0.02 (0.01-0.04) | <0.001 | <0.001 |
|  | 95 plus | 320 (1%) | 0 (0%) | 0 (0-0) | 0.918 | 0.923 |
| Social status | 1 | 1420 (4.2%) | 41 (3.8%) | 1 | (reference level) | (reference level) |
|  | 2 | 23171 (69%) | 588 (54.7%) | 0.88 (0.65-1.23) | 0.431 | 0.468 |
|  | 3 | 8756 (26.1%) | 435 (40.5%) | 1.72 (1.26-2.42) | 0.001 | 0.001 |
|  | 4 | 234 (0.7%) | 11 (1%) | 1.63 (0.79-3.1) | 0.16 | 0.19 |
| GMA Cat | 1. Very low risk | 3446 (10.3%) | 182 (16.9%) | 1 | (reference level) | (reference level) |
|  | 2. Low risk | 8319 (24.8%) | 321 (29.9%) | 0.73 (0.61-0.88) | 0.001 | 0.002 |
|  | 3. Moderate risk | 7322 (21.8%) | 239 (22.2%) | 0.62 (0.51-0.75) | <0.001 | <0.001 |
|  | 4. High risk | 8418 (25.1%) | 191 (17.8%) | 0.43 (0.35-0.53) | <0.001 | <0.001 |
|  | 5. Very high risk | 6076 (18.1%) | 142 (13.2%) | 0.44 (0.35-0.55) | <0.001 | <0.001 |
| Hypertension | 0 | 15911 (47.4%) | 563 (52.4%) | 1 | (reference level) | (reference level) |
|  | 1 | 17670 (52.6%) | 512 (47.6%) | 0.82 (0.73-0.92) | 0.001 | 0.001 |
| Neoplasm | 0 | 25733 (76.6%) | 864 (80.4%) | 1 | (reference level) | (reference level) |
|  | 1 | 7848 (23.4%) | 211 (19.6%) | 0.8 (0.69-0.93) | 0.004 | 0.005 |
| Dementia | 0 | 26565 (79.1%) | 1032 (96%) | 1 | (reference level) | (reference level) |
|  | 1 | 7016 (20.9%) | 43 (4%) | 0.16 (0.11-0.21) | <0.001 | <0.001 |
| Ictus | 0 | 27944 (83.2%) | 938 (87.3%) | 1 | (reference level) | (reference level) |
|  | 1 | 5637 (16.8%) | 137 (12.7%) | 0.72 (0.6-0.86) | <0.001 | <0.001 |
| Heart Failure | 0 | 28568 (85.1%) | 952 (88.6%) | 1 | (reference level) | (reference level) |
|  | 1 | 5013 (14.9%) | 123 (11.4%) | 0.74 (0.61-0.89) | 0.002 | 0.003 |
| Renal Insufficiency | 0 | 27858 (83%) | 931 (86.6%) | 1 | (reference level) | (reference level) |
|  | 1 | 5723 (17%) | 144 (13.4%) | 0.75 (0.63-0.9) | 0.002 | 0.002 |
| Ischemia | 0 | 29532 (87.9%) | 922 (85.8%) | 1 | (reference level) | (reference level) |
|  | 1 | 4049 (12.1%) | 153 (14.2%) | 1.21 (1.01-1.44) | 0.032 | 0.038 |
| Obesity | 0 | 25088 (74.7%) | 723 (67.3%) | 1 | (reference level) | (reference level) |
|  | 1 | 8493 (25.3%) | 352 (32.7%) | 1.44 (1.26-1.64) | <0.001 | <0.001 |
| Smoking | 0 | 31236 (93%) | 933 (86.8%) | 1 | (reference level) | (reference level) |
|  | 1 | 2345 (7%) | 142 (13.2%) | 2.03 (1.68-2.42) | <0.001 | <0.001 |
| Transplants | 0 | 33440 (99.6%) | 1061 (98.7%) | 1 | (reference level) | (reference level) |
|  | 1 | 141 (0.4%) | 14 (1.3%) | 3.13 (1.72-5.24) | <0.001 | <0.001 |
| Metabolic Syndrome | 0 | 21852 (65.1%) | 644 (59.9%) | 1 | (reference level) | (reference level) |
|  | 1 | 11729 (34.9%) | 431 (40.1%) | 1.25 (1.1-1.41) | <0.001 | <0.001 |
| Inhib Calcineurin | 0 | 33456 (99.6%) | 1061 (98.7%) | 1 | (reference level) | (reference level) |
|  | 1 | 125 (0.4%) | 14 (1.3%) | 3.53 (1.94-5.94) | <0.001 | <0.001 |
| Selective Immunosuppressants | 0 | 33382 (99.4%) | 1059 (98.5%) | 1 | (reference level) | (reference level) |
|  | 1 | 199 (0.6%) | 16 (1.5%) | 2.53 (1.46-4.1) | <0.001 | <0.001 |
| Direct Action Oral Anticoagulants | 0 | 31679 (94.3%) | 1032 (96%) | 1 | (reference level) | (reference level) |
|  | 1 | 1902 (5.7%) | 43 (4%) | 0.69 (0.5-0.93) | 0.02 | 0.05 |
| Heparins | 0 | 33089 (98.5%) | 1069 (99.4%) | 1 | (reference level) | (reference level) |
|  | 1 | 492 (1.5%) | 6 (0.6%) | 0.38 (0.15-0.77) | 0.018 | 0.024 |
| Alpha1 adrenergic inhib | 0 | 29869 (88.9%) | 922 (85.8%) | 1 | (reference level) | (reference level) |
|  | 1 | 3712 (11.1%) | 153 (14.2%) | 1.34 (1.12-1.58) | 0.001 | 0.002 |
| ARB combinations | 0 | 28049 (83.5%) | 869 (80.8%) | 1 | (reference level) | (reference level) |
|  | 1 | 5532 (16.5%) | 206 (19.2%) | 1.2 (1.03-1.4) | 0.02 | 0.03 |
| HMCoA reductase | 0 | 23904 (71.2%) | 695 (64.7%) | 1 | (reference level) | (reference level) |
|  | 1 | 9677 (28.8%) | 380 (35.3%) | 1.35 (1.19-1.53) | <0.001 | <0.001 |
| Num drugs patient |  | 1.2 (1.3) | 1.4 (1.4) | 1.09 (1.04-1.14) | <0.001 | <0.001 |
| Polypharmacy |  | 0.0036 (0.06) | 0.0074 (0.086) | 2.07 (0.93-3.98) | 0.047 | 0.052 |
| GMA: Adjusted morbidity groups. Social status: 1) exempted (non-working population or non-contributory pension recipients); 2) < 18,000€ income per year; 3) 18,000€ to 100,000€ income per year; and 4) > 100,000€ per year. ACEi: Angiotensin-converting-enzyme inhibitors, ARB: Angiotensin receptor blockers, DPP4: Dipeptidyl peptidase 4, HIV: human immunodeficiency virus, HMCoA: ß-Hydroxy ß-methylglutaryl-CoA, Inhib: inhibitors, COPD: Chronic obstructive pulmonary disease, TI: reverse transcriptase, TNF: Tumor necrosis factor, Vit K: vitamine K. The adjusted p-value was computed using the Benjamini-Yekutieli (BY) correction method. | | | | | |  |

**Supplementary Material 8. Socio-demographic and health characteristics of participants who died during hospitalisation aged 18 to 65 years in the cohort studied.** Odds Ratio (OR) estimates and 95% confidence intervals (CI) for significant socio-demographic, chronic illness, and medication variables in univariate logistic regression for the risk of death during hospitalisation in participants diagnosed with COVID-19. Data are presented as the total number of participants and the percentage (%) in relation to the overall cohort for categorical variables, and mean and standard deviation (SD) for numerical variables, using a univariate model by age groups (18 to 65 years old). Probabilities were calculated using the Student’s t-test for continuous variables and chi-squared for categorical variables (N = 10,511). The reference group for disease and drug variables was “No”.

| **Variable** | **Levels** | **Non exitus in hospitalisation 10127 (96.35%)** | **Exitus in hospitalisation 384 (3.65%)** | **OR (CI)** | **Pr(>\|z\|)** | **Adjusted-p-val BY** |
| --- | --- | --- | --- | --- | --- | --- |
| Sex | Female | 4349 (42.9%) | 113 (29.4%) | 1 | (reference level) | (reference level) |
|  | Male | 5778 (57.1%) | 271 (70.6%) | 1.81 (1.45-2.26) | <0.001 | <0.001 |
| Age at exposure | 20-24 | 202 (2%) | 2 (0.5%) | 1 | (reference level) | (reference level) |
|  | 25-29 | 334 (3.3%) | 3 (0.8%) | 0.91 (0.15-6.93) | 0.915 | 0.981 |
|  | 30-34 | 445 (4.4%) | 2 (0.5%) | 0.45 (0.05-3.8) | 0.431 | 0.45 |
|  | 35-39 | 725 (7.2%) | 4 (1%) | 0.56 (0.11-4.04) | 0.501 | 0.512 |
|  | 40-44 | 1099 (10.9%) | 19 (4.9%) | 1.75 (0.5-11.01) | 0.456 | 0.488 |
|  | 45-49 | 1485 (14.7%) | 21 (5.5%) | 1.43 (0.41-8.97) | 0.632 | 0.698 |
|  | 50-54 | 1800 (17.8%) | 56 (14.6%) | 3.14 (0.97-19.27) | 0.114 | 0.145 |
|  | 55-59 | 2023 (20%) | 107 (27.9%) | 5.34 (1.68-32.53) | 0.02 | 0.026 |
|  | 60-64 | 2014 (19.9%) | 170 (44.3%) | 8.53 (2.7-51.77) | 0.003 | 0.003 |
| Social status | 1 | 531 (5.2%) | 43 (11.2%) | 1 | (reference level) | (reference level) |
|  | 2 | 5855 (57.8%) | 223 (58.1%) | 0.47 (0.34-0.67) | <0.001 | <0.001 |
|  | 3 | 3639 (35.9%) | 117 (30.5%) | 0.4 (0.28-0.58) | <0.001 | <0.001 |
|  | 4 | 102 (1%) | 1 (0.3%) | 0.12 (0.01-0.57) | 0.038 | 0.045 |
| GMA Cat | 1. Very low risk | 3421 (33.8%) | 82 (21.4%) | 1 | (reference level) | (reference level) |
|  | 2. Low risk | 2824 (27.9%) | 89 (23.2%) | 1.31 (0.97-1.78) | 0.078 | 0.088 |
|  | 3. Moderate risk | 1700 (16.8%) | 83 (21.6%) | 2.04 (1.49-2.78) | <0.001 | <0.001 |
|  | 4. High risk | 1397 (13.8%) | 64 (16.7%) | 1.91 (1.37-2.66) | <0.001 | <0.001 |
|  | 5. Very high risk | 785 (7.8%) | 66 (17.2%) | 3.51 (2.51-4.89) | <0.001 | <0.001 |
| Diabetes | 0 | 8923 (88.1%) | 296 (77.1%) | 1 | (reference level) | (reference level) |
|  | 1 | 1204 (11.9%) | 88 (22.9%) | 2.2 (1.71-2.8) | <0.001 | <0.001 |
| Hypertension | 0 | 8130 (80.3%) | 266 (69.3%) | 1 | (reference level) | (reference level) |
|  | 1 | 1997 (19.7%) | 118 (30.7%) | 1.81 (1.44-2.25) | <0.001 | <0.001 |
| Neoplasm | 0 | 9416 (93%) | 308 (80.2%) | 1 | (reference level) | (reference level) |
|  | 1 | 711 (7%) | 76 (19.8%) | 3.27 (2.5-4.23) | <0.001 | <0.001 |
| Dementia | 0 | 10042 (99.2%) | 375 (97.7%) | 1 | (reference level) | (reference level) |
|  | 1 | 85 (0.8%) | 9 (2.3%) | 2.84 (1.32-5.38) | 0.003 | 0.004 |
| Ictus | 0 | 9790 (96.7%) | 358 (93.2%) | 1 | (reference level) | (reference level) |
|  | 1 | 337 (3.3%) | 26 (6.8%) | 2.11 (1.36-3.13) | <0.001 | <0.001 |
| Heart Failure | 0 | 9959 (98.3%) | 353 (91.9%) | 1 | (reference level) | (reference level) |
|  | 1 | 168 (1.7%) | 31 (8.1%) | 5.21 (3.44-7.64) | <0.001 | <0.001 |
| Renal Insufficiency | 0 | 9871 (97.5%) | 348 (90.6%) | 1 | (reference level) | (reference level) |
|  | 1 | 256 (2.5%) | 36 (9.4%) | 3.99 (2.73-5.67) | <0.001 | <0.001 |
| Ischemia | 0 | 9757 (96.3%) | 341 (88.8%) | 1 | (reference level) | (reference level) |
|  | 1 | 370 (3.7%) | 43 (11.2%) | 3.33 (2.35-4.59) | <0.001 | <0.001 |
| COPD | 0 | 9807 (96.8%) | 332 (86.5%) | 1 | (reference level) | (reference level) |
|  | 1 | 320 (3.2%) | 52 (13.5%) | 4.8 (3.48-6.51) | <0.001 | <0.001 |
| Hematological Neoplasm | 0 | 10070 (99.4%) | 378 (98.4%) | 1 | (reference level) | (reference level) |
|  | 1 | 57 (0.6%) | 6 (1.6%) | 2.8 (1.08-6.04) | 0.017 | 0.021 |
| Obesity | 0 | 7755 (76.6%) | 248 (64.6%) | 1 | (reference level) | (reference level) |
|  | 1 | 2372 (23.4%) | 136 (35.4%) | 1.79 (1.44-2.22) | <0.001 | <0.001 |
| Smoking | 0 | 9263 (91.5%) | 311 (81%) | 1 | (reference level) | (reference level) |
|  | 1 | 864 (8.5%) | 73 (19%) | 2.52 (1.92-3.26) | <0.001 | <0.001 |
| Transplants | 0 | 10055 (99.3%) | 370 (96.4%) | 1 | (reference level) | (reference level) |
|  | 1 | 72 (0.7%) | 14 (3.6%) | 5.28 (2.84-9.16) | <0.001 | <0.001 |
| Metabolic Syndrome | 0 | 8216 (81.1%) | 270 (70.3%) | 1 | (reference level) | (reference level) |
|  | 1 | 1911 (18.9%) | 114 (29.7%) | 1.82 (1.45-2.27) | <0.001 | <0.001 |
| Inhib Calcineurin | 0 | 10045 (99.2%) | 365 (95.1%) | 1 | (reference level) | (reference level) |
|  | 1 | 82 (0.8%) | 19 (4.9%) | 6.38 (3.72-10.39) | <0.001 | <0.001 |
| Rituximab | 0 | 10111 (99.8%) | 381 (99.2%) | 1 | (reference level) | (reference level) |
|  | 1 | 16 (0.2%) | 3 (0.8%) | 4.98 (1.15-15.01) | 0.011 | 0.015 |
| Selective Immunosuppressants | 0 | 10027 (99%) | 363 (94.5%) | 1 | (reference level) | (reference level) |
|  | 1 | 100 (1%) | 21 (5.5%) | 5.8 (3.49-9.2) | <0.001 | <0.001 |
| Systemic Corticosteroids | 0 | 9850 (97.3%) | 340 (88.5%) | 1 | (reference level) | (reference level) |
|  | 1 | 277 (2.7%) | 44 (11.5%) | 4.6 (3.25-6.37) | <0.001 | <0.001 |
| Metformin | 0 | 9706 (95.8%) | 353 (91.9%) | 1 | (reference level) | (reference level) |
|  | 1 | 421 (4.2%) | 31 (8.1%) | 2.02 (1.36-2.91) | <0.001 | <0.001 |
| Inhib DPP4 | 0 | 10064 (99.4%) | 377 (98.2%) | 1 | (reference level) | (reference level) |
|  | 1 | 63 (0.6%) | 7 (1.8%) | 2.97 (1.23-6.08) | 0.007 | 0.009 |
| Direct Action Oral Anticoagulants | 0 | 10077 (99.5%) | 378 (98.4%) | 1 | (reference level) | (reference level) |
|  | 1 | 50 (0.5%) | 6 (1.6%) | 3.2 (1.22-6.94) | 0.008 | 0.01 |
| VitK Antagonists | 0 | 10053 (99.3%) | 375 (97.7%) | 1 | (reference level) | (reference level) |
|  | 1 | 74 (0.7%) | 9 (2.3%) | 3.26 (1.51-6.22) | 0.001 | 0.002 |
| Alpha1 adrenergic inhib | 0 | 9900 (97.8%) | 348 (90.6%) | 1 | (reference level) | (reference level) |
|  | 1 | 227 (2.2%) | 36 (9.4%) | 4.51 (3.08-6.43) | <0.001 | <0.001 |
| ACEi combinations | 0 | 9206 (90.9%) | 329 (85.7%) | 1 | (reference level) | (reference level) |
|  | 1 | 921 (9.1%) | 55 (14.3%) | 1.67 (1.23-2.22) | 0.001 | 0.002 |
| ARB combinations | 0 | 9588 (94.7%) | 351 (91.4%) | 1 | (reference level) | (reference level) |
|  | 1 | 539 (5.3%) | 33 (8.6%) | 1.67 (1.14-2.38) | 0.006 | 0.008 |
| HMCoA reductase | 0 | 9200 (90.8%) | 300 (78.1%) | 1 | (reference level) | (reference level) |
|  | 1 | 927 (9.2%) | 84 (21.9%) | 2.78 (2.15-3.56) | <0.001 | <0.001 |
| Num drugs patient |  | 0.46 (0.92) | 1 (1.4) | 1.52 (1.41-1.63) | <0.001 | <0.001 |
| Polypharmacy |  | 0.0014 (0.037) | 0.0078 (0.088) | 5.69 (1.31-17.51) | 0.006 | 0.007 |
| GMA: Adjusted morbidity groups. Social status: 1) exempted (non-working population or non-contributory pension recipients); 2) < 18,000€ income per year; 3) 18,000€ to 100,000€ income per year; and 4) > 100,000€ per year. ACEi: Angiotensin-converting-enzyme inhibitors, ARB: Angiotensin receptor blockers, DPP4: Dipeptidyl peptidase 4, HIV: human immunodeficiency virus, HMCoA: ß-Hydroxy ß-methylglutaryl-CoA, Inhib: inhibitors, COPD: Chronic obstructive pulmonary disease, TI: reverse transcriptase, TNF: Tumor necrosis factor, Vit K: vitamine K. The adjusted p-value was computed using the Benjamini-Yekutieli (BY) correction method. | | | | | |  |

**Supplementary Material 9. Socio-demographic and health characteristics of participants who died during hospitalisation over 65 years in the cohort studied.** Odds Ratio (OR) estimates and 95% confidence intervals (CI) for significant socio-demographic, chronic illness, and medication variables in univariate logistic regression for the risk of death during hospitalisation in participants diagnosed with COVID-19. Data are presented as the total number of participants and the percentage (%) in relation to the overall cohort for categorical variables, and mean and standard deviation (SD) for numerical variables, using a univariate model by age groups (18 to 65 years old). Probabilities were calculated using the Student’s t-test for continuous variables and chi-squared for categorical variables (N = 11,934). The reference group for disease and drug variables was “No”.

| **Variable** | **Levels** | **Non exitus in hospitalisation 8931 (74.84%)** | **Exitus in hospitalisation 3003 (25.16%)** | **OR (CI)** | **Pr(>\|z\|)** | **Adjusted–p-val BY** |
| --- | --- | --- | --- | --- | --- | --- |
| Sex | Female | 4483 (50.2%) | 1299 (43.3%) | 1 | (reference level) | (reference level) |
|  | Male | 4448 (49.8%) | 1704 (56.7%) | 1.32 (1.22-1.44) | <0.001 | <0.001 |
| Age at exposure | 65-69 | 1867 (20.9%) | 283 (9.4%) | 1 | (reference level) | (reference level) |
|  | 70-74 | 1965 (22%) | 417 (13.9%) | 1.4 (1.19-1.65) | <0.001 | <0.001 |
|  | 75-79 | 1831 (20.5%) | 618 (20.6%) | 2.23 (1.91-2.6) | <0.001 | <0.001 |
|  | 80-84 | 1436 (16.1%) | 692 (23%) | 3.18 (2.73-3.71) | <0.001 | <0.001 |
|  | 85-89 | 1322 (14.8%) | 694 (23.1%) | 3.46 (2.97-4.05) | <0.001 | <0.001 |
|  | 90-94 | 487 (5.5%) | 276 (9.2%) | 3.74 (3.08-4.54) | <0.001 | <0.001 |
|  | 95 plus | 23 (0.3%) | 23 (0.8%) | 6.6 (3.64-11.96) | <0.001 | <0.001 |
| Social status | 1 | 352 (3.9%) | 105 (3.5%) | 1 | (reference level) | (reference level) |
|  | 2 | 5817 (65.1%) | 2044 (68.1%) | 1.18 (0.95-1.48) | 0.151 | 0.164 |
|  | 3 | 2708 (30.3%) | 840 (28%) | 1.04 (0.83-1.32) | 0.74 | 0.82 |
|  | 4 | 54 (0.6%) | 14 (0.5%) | 0.87 (0.45-1.59) | 0.661 | 0.691 |
| GMA Cat | 1. Very low risk | 1020 (11.4%) | 213 (7.1%) | 1 | (reference level) | (reference level) |
|  | 2. Low risk | 2416 (27.1%) | 612 (20.4%) | 1.21 (1.02-1.44) | 0.028 | 0.035 |
|  | 3. Moderate risk | 1938 (21.7%) | 645 (21.5%) | 1.59 (1.34-1.9) | <0.001 | <0.001 |
|  | 4. High risk | 2093 (23.4%) | 812 (27%) | 1.86 (1.57-2.2) | <0.001 | <0.001 |
|  | 5. Very high risk | 1464 (16.4%) | 721 (24%) | 2.36 (1.99-2.81) | <0.001 | <0.001 |
| Diabetes | 0 | 6493 (72.7%) | 2032 (67.7%) | 1 | (reference level) | (reference level) |
|  | 1 | 2438 (27.3%) | 971 (32.3%) | 1.27 (1.16-1.39) | <0.001 | <0.001 |
| Hypertension | 0 | 4322 (48.4%) | 1308 (43.6%) | 1 | (reference level) | (reference level) |
|  | 1 | 4609 (51.6%) | 1695 (56.4%) | 1.22 (1.12-1.32) | <0.001 | <0.001 |
| Neoplasm | 0 | 6715 (75.2%) | 2135 (71.1%) | 1 | (reference level) | (reference level) |
|  | 1 | 2216 (24.8%) | 868 (28.9%) | 1.23 (1.12-1.35) | <0.001 | <0.001 |
| Dementia | 0 | 7779 (87.1%) | 2449 (81.6%) | 1 | (reference level) | (reference level) |
|  | 1 | 1152 (12.9%) | 554 (18.4%) | 1.53 (1.37-1.71) | <0.001 | <0.001 |
| Ictus | 0 | 7638 (85.5%) | 2420 (80.6%) | 1 | (reference level) | (reference level) |
|  | 1 | 1293 (14.5%) | 583 (19.4%) | 1.42 (1.28-1.58) | <0.001 | <0.001 |
| Heart Failure | 0 | 7659 (85.8%) | 2364 (78.7%) | 1 | (reference level) | (reference level) |
|  | 1 | 1272 (14.2%) | 639 (21.3%) | 1.63 (1.46-1.81) | <0.001 | <0.001 |
| Renal Insufficiency | 0 | 7519 (84.2%) | 2312 (77%) | 1 | (reference level) | (reference level) |
|  | 1 | 1412 (15.8%) | 691 (23%) | 1.59 (1.44-1.76) | <0.001 | <0.001 |
| Ischemia | 0 | 7755 (86.8%) | 2527 (84.1%) | 1 | (reference level) | (reference level) |
|  | 1 | 1176 (13.2%) | 476 (15.9%) | 1.24 (1.11-1.39) | <0.001 | <0.001 |
| COPD | 0 | 7614 (85.3%) | 2412 (80.3%) | 1 | (reference level) | (reference level) |
|  | 1 | 1317 (14.7%) | 591 (19.7%) | 1.42 (1.27-1.58) | <0.001 | <0.001 |
| Hematological Neoplasm | 0 | 8816 (98.7%) | 2949 (98.2%) | 1 | (reference level) | (reference level) |
|  | 1 | 115 (1.3%) | 54 (1.8%) | 1.4 (1.01-1.93) | 0.041 | 0.048 |
| Inhib Calcineurin | 0 | 8876 (99.4%) | 2973 (99%) | 1 | (reference level) | (reference level) |
|  | 1 | 55 (0.6%) | 30 (1%) | 1.63 (1.03-2.53) | 0.032 | 0.074 |
| Selective Immunosuppressants | 0 | 8850 (99.1%) | 2962 (98.6%) | 1 | (reference level) | (reference level) |
|  | 1 | 81 (0.9%) | 41 (1.4%) | 1.51 (1.03-2.19) | 0.032 | 0.076 |
| Systemic Corticosteroids | 0 | 8466 (94.8%) | 2781 (92.6%) | 1 | (reference level) | (reference level) |
|  | 1 | 465 (5.2%) | 222 (7.4%) | 1.45 (1.23-1.71) | <0.001 | <0.001 |
| Inhib DPP4 | 0 | 8625 (96.6%) | 2865 (95.4%) | 1 | (reference level) | (reference level) |
|  | 1 | 306 (3.4%) | 138 (4.6%) | 1.36 (1.1-1.66) | 0.004 | 0.005 |
| Direct Action Oral Anticoagulants | 0 | 8455 (94.7%) | 2793 (93%) | 1 | (reference level) | (reference level) |
|  | 1 | 476 (5.3%) | 210 (7%) | 1.34 (1.13-1.58) | 0.001 | 0.003 |
| VitK Antagonists | 0 | 8451 (94.6%) | 2785 (92.7%) | 1 | (reference level) | (reference level) |
|  | 1 | 480 (5.4%) | 218 (7.3%) | 1.38 (1.17-1.62) | <0.001 | <0.001 |
| Heparins | 0 | 8762 (98.1%) | 2965 (98.7%) | 1 | (reference level) | (reference level) |
|  | 1 | 169 (1.9%) | 38 (1.3%) | 0.66 (0.46-0.94) | 0.024 | 0.029 |
| Alpha1 adrenergic inhib | 0 | 7716 (86.4%) | 2491 (83%) | 1 | (reference level) | (reference level) |
|  | 1 | 1215 (13.6%) | 512 (17%) | 1.31 (1.17-1.46) | <0.001 | <0.001 |
| HMCoA reductase | 0 | 5953 (66.7%) | 1952 (65%) | 1 | (reference level) | (reference level) |
|  | 1 | 2978 (33.3%) | 1051 (35%) | 1.08 (0.99-1.17) | 0.097 | 0.12 |
| Num drugs patient |  | 1.1 (1.2) | 1.5 (1.4) | 1.07 (1.04-1.11) | <0.001 | <0.001 |
| GMA: Adjusted morbidity groups. Social status: 1) exempted (non-working population or non-contributory pension recipients); 2) < 18,000€ income per year; 3) 18,000€ to 100,000€ income per year; and 4) > 100,000€ per year. ACEi: Angiotensin-converting-enzyme inhibitors, ARB: Angiotensin receptor blockers, DPP4: Dipeptidyl peptidase 4, HIV: human immunodeficiency virus, HMCoA: ß-Hydroxy ß-methylglutaryl-CoA, Inhib: inhibitors, COPD: Chronic obstructive pulmonary disease, TI: reverse transcriptase, TNF: Tumor necrosis factor, Vit K: vitamine K. The adjusted p-value was computed using the Benjamini-Yekutieli (BY) correction method. | | | | | |  |

**Supplementary Material 10. Boxplot comparison of ROC AUC scores for XGBoost, KNN, SVM, and AdaBoost models.** Distribution (median, interquartile range, and outliers) of ROCAUC values obtained from repeated model evaluations, highlighting the superior performance of XGBoost relative to the other methods. a) risk of hospitalisation, b) risk of mortality, c) risk of ICU admission in patients 18 to 65 years old; d) risk of hospitalisation, e) risk of mortality, f) risk of ICU admission in patients older than 65 years old. Models are ranked from better to worst performing based on ROCAUC metrics.

**
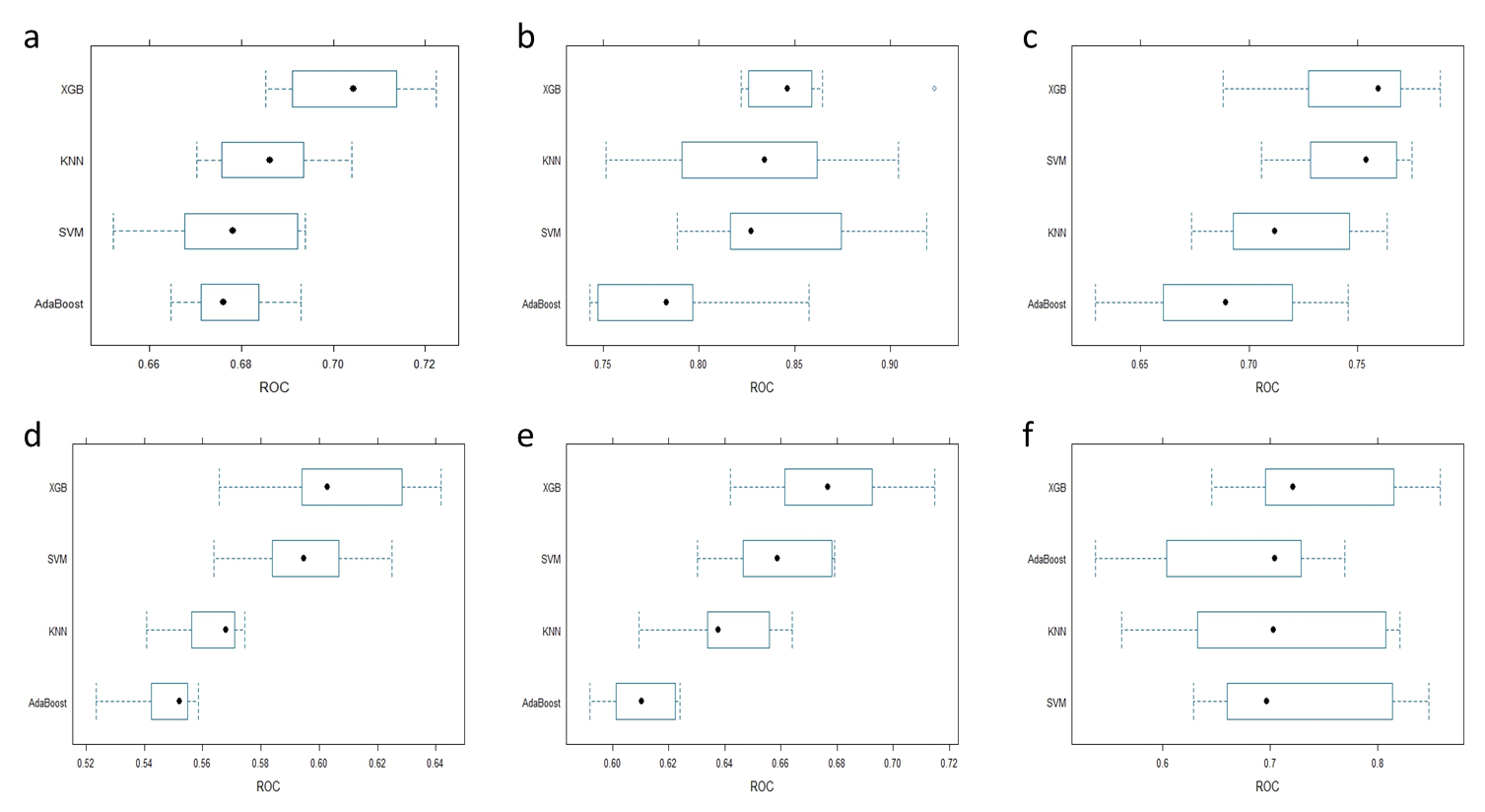
**

**Supplementary Material 11. Performance measures for the co-existing diseases models evaluated in participants 18 to 65, and above 65.** XGBoost models for comorbidity features were built for the 3 outcomes: a) risk of hospitalisation from COVID-19, b) risk of mortality, c) risk of ICU admission, in participants aged 18 to 65 years old; and d) risk of hospitalisation e) risk of mortality, and f) risk of ICU admission in participants older than 65 years.

**
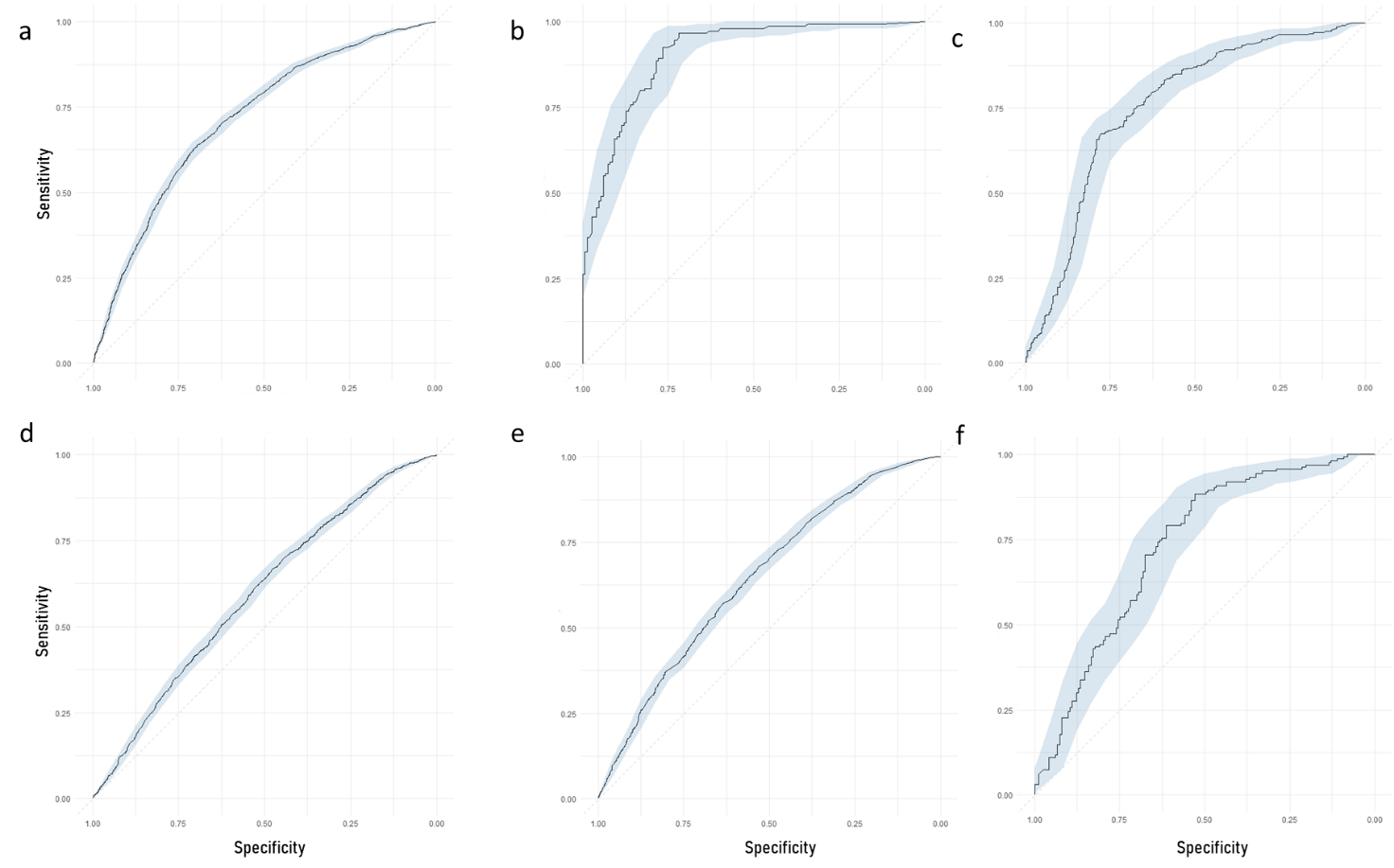
**

**Supplementary Material 12. Variable importance (SHAP values) for the XGBoost comorbidities model to determine the risk of hospitalisation from COVID-19 in participants by age groups**: a) from 18 to 65 years old, and b) above 65 years old, for the comorbidities model. High SHAP values mean a higher probability of hospitalisation. Purple represents high variable values and yellow represents lower variable values (in the categorical variables one-hot-encoded, it is 0 yellow and 1 purple). Each point represents an instance (participant) for that variable in the dataset. COPD: Chronic obstructive pulmonary disease.

**a**

**
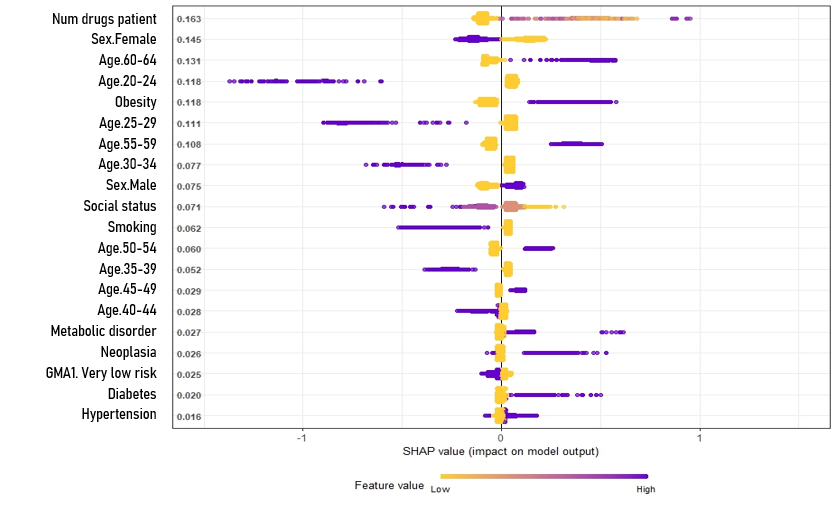
**

**b**

**
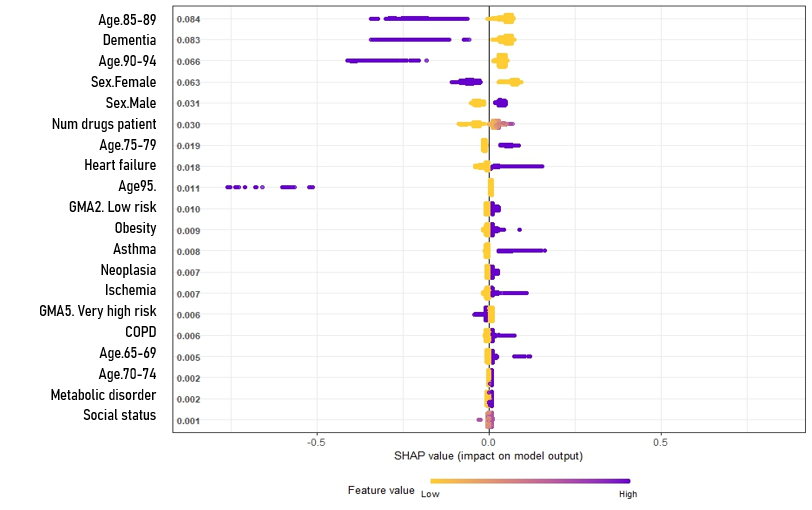
**

**Supplementary Material 13. Variable importance (SHAP values) for the XGBoost comorbidities model to determine the risk of mortality from COVID-19 in participants by age groups**: a) from 18 to 65 years old, and b) above 65 years old, for the comorbidities model. High SHAP values mean a higher probability of mortality. Purple represents high variable values and yellow represents lower variable values (in the categorical variables one-hot-encoded, it is 0 yellow and 1 purple). Each point represents an instance (participant) for that variable in the dataset. COPD: Chronic obstructive pulmonary disease.

**a**

**
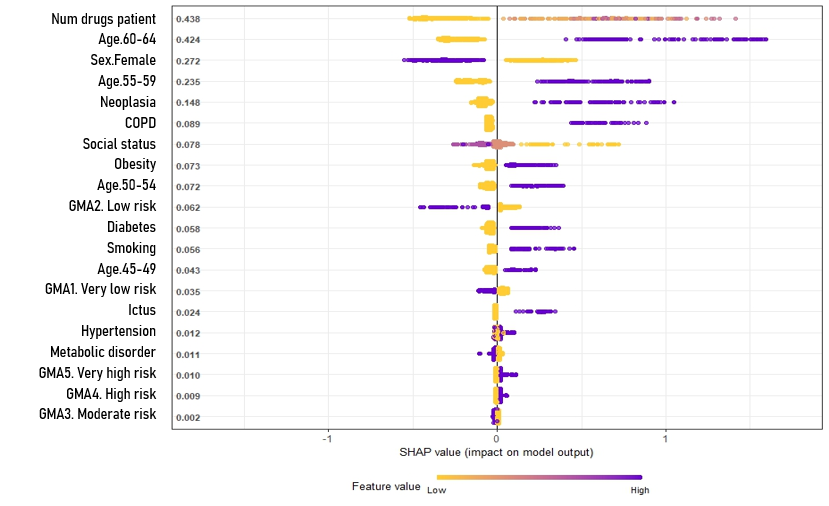
**

**b**

**
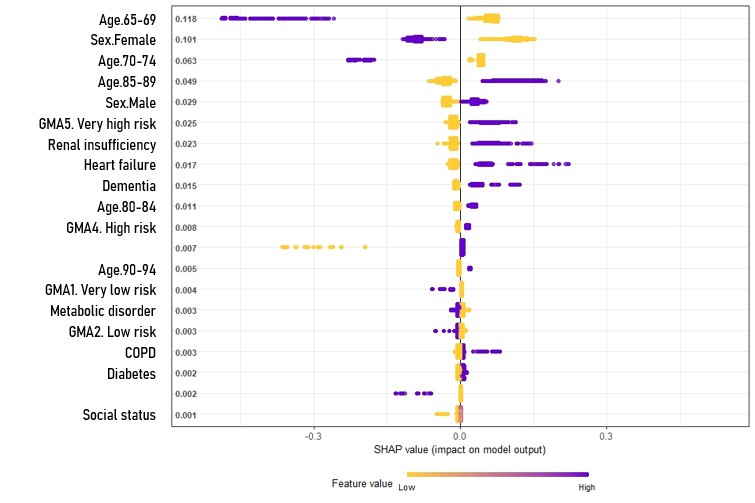
**

**Supplementary Material 14. Variable importance (SHAP values) for the XGBoost comorbidities model to determine the risk of ICU admission from COVID-19 in participants by age groups**: a) from 18 to 65 years old, and b) above 65 years old, for the comorbidities model. High SHAP values mean a higher probability of ICU admission. Purple represents high variable values and yellow represents lower variable values (in the categorical variables one-hot-encoded, it is 0 yellow and 1 purple). Each point represents an instance (participant) for that variable in the dataset. COPD: Chronic obstructive pulmonary disease.

**a**

**
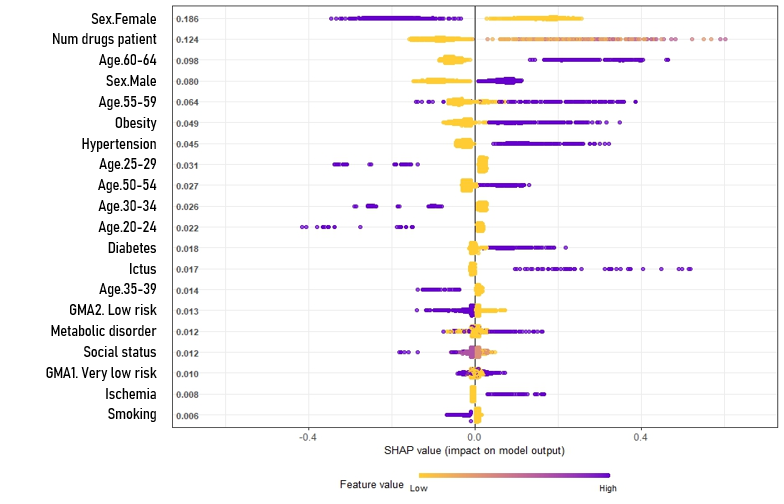
**

**b**

**
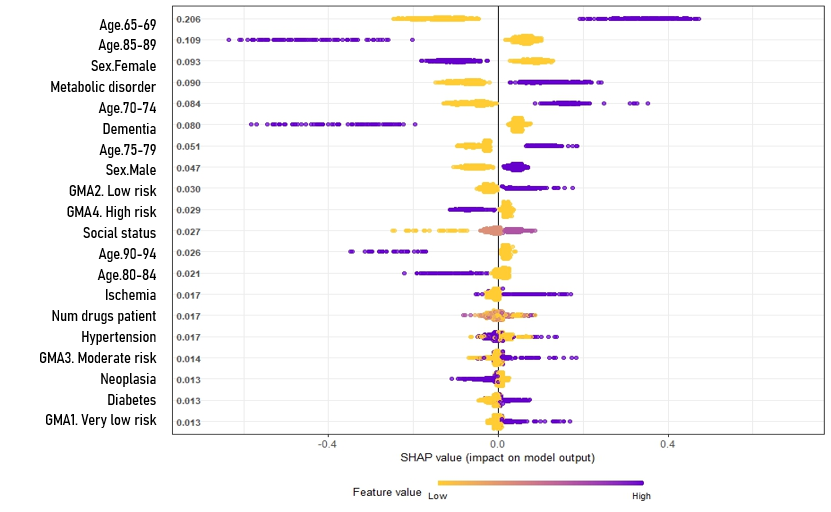
**

**Supplementary Material 15.** Local explanation for sampled participants 65 years old or below using the XGBoost model for risk of hospitalisation. The sign (and colors) indicates what increases the odds (yellow color) and what decreases the odds (red maroon) of hospitalisation. The number and length of the bar indicates the size of the effect of each added variable in the final prediction (probability above the last bar).

**Participant 1**

**
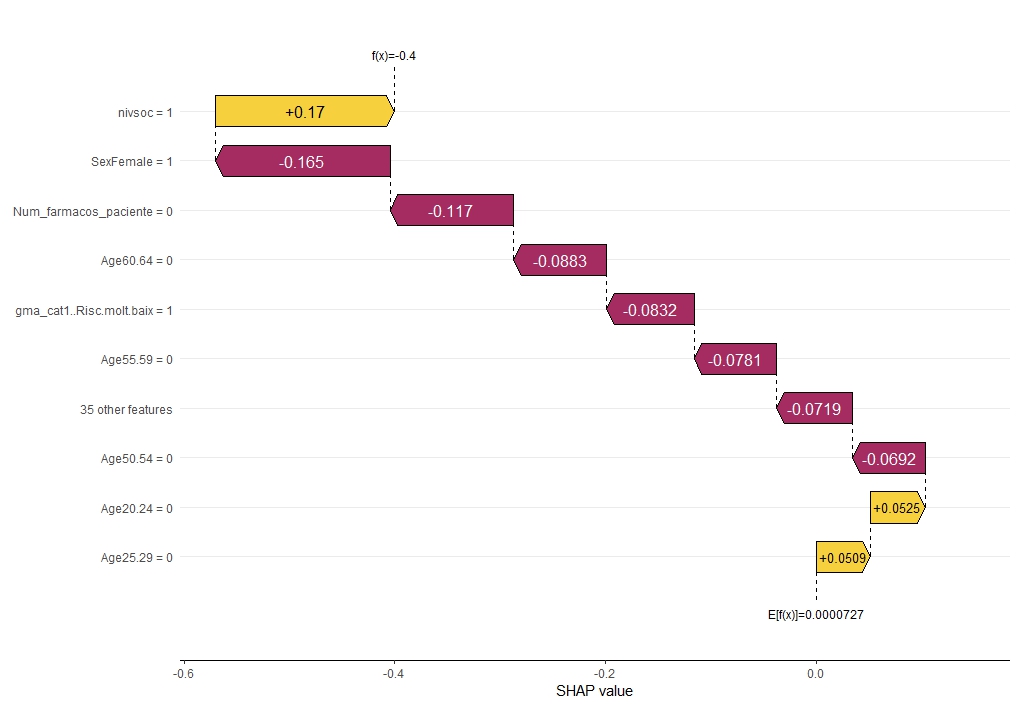
**

**Participant 2**

**
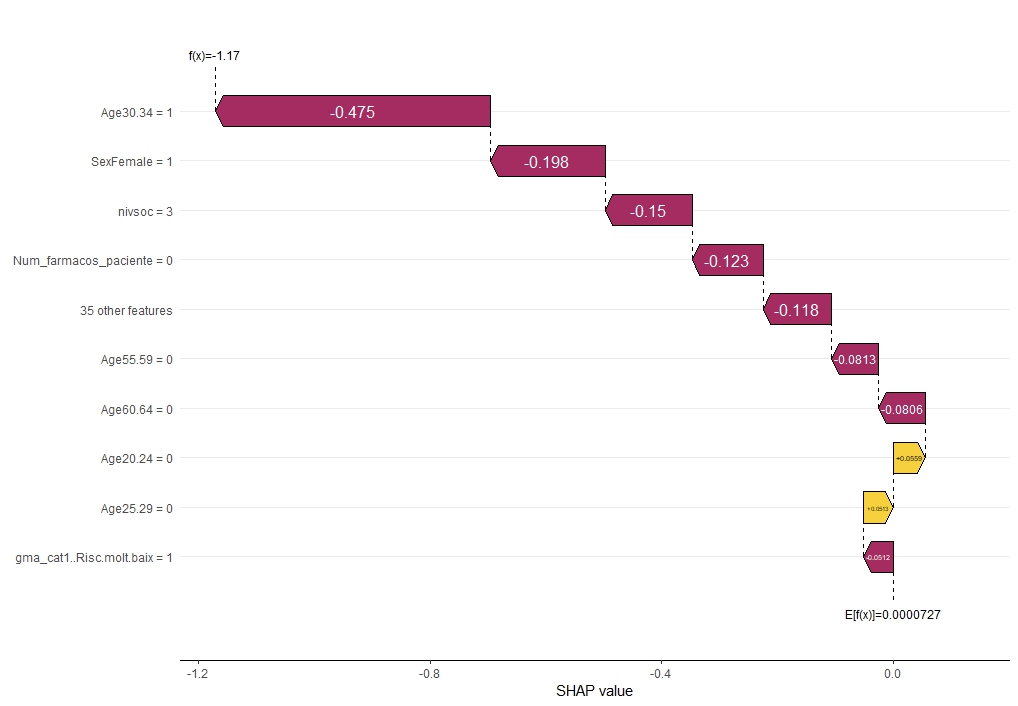
**

**Participant 3**

**
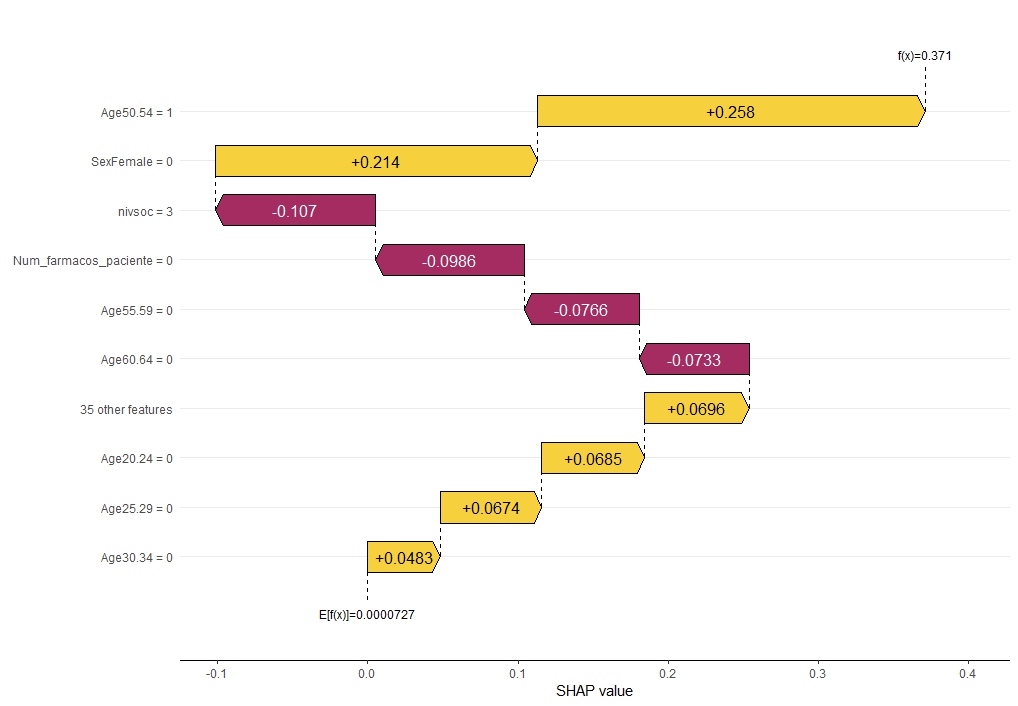
**

**Supplementary Material 16.** Multivariate analysis for the risk of hospitalization in participants 65 years of age or below (left ) and over 65 (right) stratified by relevant risk factors and drugs of interest for the comorbidities (model adjusted for age and sex). Hypertension drugs were ACEi, and ARBs, Diabetes drugs were Biguanides and DPP4, and Metabolic disorder drugs were statins.

|  | **Participants 18 to 65 years** | | | | | | | **Participants over 65 years** | | | | | |  |
| --- | --- | --- | --- | --- | --- | --- | --- | --- | --- | --- | --- | --- | --- | --- |
| **Variable** | **Levels** | **aOR** | **CI (lower)** | **CI (upper)** | **Pr(>\|z\|)** | **N** | **total N** | **Levels** | **OR** | **CI (lower)** | **CI (upper)** | **Pr(>\|z\|)** | **N=** | **total N** |
| **Drugs** | **ACEi** | 1 | 0.85 | 1.13 | 0.8 | 370 | 1572 | **ACEi** | 1.2 | 1.07 | 1.35 | 0.001 | 693 | 3842 |
|  | **ARB** | 1.1 | 0.89 | 1.27 | 0.5 | 203 |  | **ARB** | 1.2 | 1.04 | 1.36 | 0.009 | 466 |  |
|  | **ARB (ref ACEi)** | 1.1 | 0.89 | 1.32 | 0.4 |  |  | **ARB (ref ACEi)** | 1 | 0.86 | 1.14 | 0.9 |  |  |
|  | **Biguanides** | 1 | 0.87 | 1.2 | 0.8 | 443 | 1260 | **Biguanides** | 1.2 | 1.04 | 1.35 | <0.001 | 1331 | 3327 |
|  | **DPP4** | 1 | 0.62 | 1.51 | 0.9 | 30 |  | **DPP4** | 1.5 | 1.27 | 1.88 | <0.001 | 253 |  |
|  | **Biguanides (ref DPP4)** | 1 | 0.68 | 1.64 | 0.9 |  |  | **Biguanides (ref DPP4)** | 0.8 | 0.65 | 0.92 | 0.004 |  |  |
|  | **Statins** | 1.1 | 0.95 | 1.35 | 0.2 | 193 | 1873 | **Statins** | 1.2 | 1.05 | 1.37 | 0.009 | 484 | 3830 |

**Supplementary Material 17.** Multivariate analysis for the risk of mortality in participants 65 years of age or below (left ) and over 65 (right) stratified by relevant risk factors and drugs of interest for the comorbidities (model adjusted for age and sex). Hypertension drugs were ACEi, and ARBs, Diabetes drugs were Biguanides and DPP4, and Metabolic disorder drugs were statins.

|  | **Participants 18 to 65 years** | | | | | | | **Participants over 65 years** | | | | | |  |
| --- | --- | --- | --- | --- | --- | --- | --- | --- | --- | --- | --- | --- | --- | --- |
| **Variable** | **Levels** | **aOR** | **CI (lower)** | **CI (upper)** | **Pr(>\|z\|)** | **N** | **total N** | **Levels** | **OR** | **CI (lower)** | **CI (upper)** | **Pr(>\|z\|)** | **N** | **total N** |
| **Drugs** | **ACEi** | 0.9 | 0.52 | 1.55 | 0.7 | 20 |  | **ACEi** | **0.8** | 0.68 | 0.9 | <0.001 | 317 | 2228 |
|  | **ARB** | 0.9 | 0.43 | 1.7 | 0.7 | 11 |  | **ARB** | **0.8** | 0.68 | 0.95 | 0.009 | 211 |  |
|  | **ARB (ref ACEi)** | 1 | 0.45 | 2.03 | >0.9 |  |  | **ARB (ref ACEi)** | **1** | 0.84 | 1.24 | 0.8 |  |  |
|  | **Biguanides** | 1.3 | 0.78 | 2.24 | 0.3 | 42 |  | **Biguanides** | **0.8** | 0.68 | 0.92 | 0.003 | 646 | 1884 |
|  | **DPP4** | 3 | 1.17 | 7 | 0.014 | 7 |  | **DPP4** | **1.2** | 0.96 | 1.49 | 0.1 | 162 |  |
|  | **Biguanides (ref DPP4)** | 0.4 | 0.18 | 0.99 | 0.048 |  |  | **Biguanides (ref DPP4)** | **0.7** | 0.54 | 0.81 | <0.001 |  |  |
|  | **Statins** | 2.1 | 1.19 | 3.66 | 0.008 | 18 |  | **Statins** | **1** | 0.85 | 1.21 | 0.9 | 204 |  |

**Supplementary Material 18.** Multivariate analysis for the risk of ICU admission in participants 65 years of age or below (left ) and over 65 (right) stratified by relevant risk factors and drugs of interest for the comorbidities (model adjusted for age and sex). Hypertension drugs were ACEi, and ARBs, Diabetes drugs were Biguanides and DPP4, and Metabolic disorder drugs were statins.

|  | **Participants 18 to 65 years** | | | | | | | **Participants over 65 years** | | | | | |  |
| --- | --- | --- | --- | --- | --- | --- | --- | --- | --- | --- | --- | --- | --- | --- |
| **Variable** | **Levels** | **aOR** | **CI (lower)** | **CI (upper)** | **Pr(>\|z\|)** | **N** | **total N** | **Levels** | **OR** | **CI (lower)** | **CI (upper)** | **Pr(>\|z\|)** | **N** | **total N** |
| **Drugs** | **ACEi** | 0.9 | 0.6 | 1.18 | 0.3 | 52 | 243 | **ACEi** | 1.2 | 0.82 | 1.62 | 0.4 | 57 | 291 |
|  | **ARB** | 1 | 0.68 | 1.53 | 0.9 | 33 |  | **ARB** | 1.3 | 0.9 | 1.9 | 0.14 | 45 |  |
|  | **ARB (ref ACEi)** | 1.2 | 0.77 | 1.89 | 0.4 |  |  | **ARB (ref ACEi)** | 1.1 | 0.76 | 1.17 | 0.5 |  |  |
|  | **Biguanides** | 0.8 | 0.57 | 1.13 | 0.2 | 69 | 224 | **Biguanides** | 0.9 | 0.64 | 1.27 | 0.5 | 126 | 310 |
|  | **DPP4** | 0.3 | 0.05 | 1.03 | 0.11 | 2 |  | **DPP4** | 1 | 0.54 | 1.66 | 0.9 | 18 |  |
|  | **Biguanides (ref DPP4)** | 2.5 | 0.78 | 15.6 | 0.2 |  |  | **Biguanides (ref DPP4)** | 0.9 | 0.57 | 1.6 | 0.8 |  |  |
|  | **Statins** | 1.1 | 0.74 | 1.71 | 0.5 | 28 | 281 | **Statins** | 0.7 | 0.51 | 1.04 | 0.089 | 45 | 398 |
